# Synergistically engineered Ag@NiO–TiO_2_ ternary nanocomposites as dual-functional photocatalysts for pollutant degradation and antibacterial applications

**DOI:** 10.1039/d6ra06547c

**Published:** 2026-09-29

**Authors:** Iftikhar Ahmad, Muhammad Tahir

**Affiliations:** a Chemical and Petroleum Engineering Department, United Arab Emirates University P.O. BOX 15551 Al Ain United Arab Emirates muhammad.tahir@uaeu.ac.ae/bttahir@yahoo.com

## Abstract

A synergistically engineered Ag@NiO–TiO_2_ ternary nanocomposite was synthesized by a hydrothermal method, and its performance was evaluated by organic pollutant degradation and antibiotic removal. The photocatalytic degradation of methylene blue, (MB), crystal violet (CV), and ciprofloxacin (CIP) by the nanocomposite under UV-visible light irradiation was systematically studied for comparison. The photocatalytic removal achieved by the Ag@NiO–TiO_2_ nanocomposite for MB and CV was 88% and 90%, respectively, under UV-visible light irradiation after 1 h. Similarly, 57% removal of CIP from water was achieved using 20 mg L^−1^ of solution after 180 minutes of UV-visible light irradiation. The superior UV-visible photocatalytic capacity of the nanocomposite toward organic dyes was confirmed by its high removal efficiencies for MB and CV, and its potential for treating pharmaceutical wastewater was revealed by the moderate removal of CIP. The prepared compound was also evaluated for antimicrobial activities, and significant antimicrobial activities were achieved against bacterial and fungal strains. The results of the Ag@NiO–TiO_2_ ternary heterojunction nanocomposite showed that it is an effective photocatalyst for degrading organic pollutants under UV-visible light irradiation. Furthermore, the photocatalyst showed enhanced antimicrobial activity against all strains. Therefore, the synthesized Ag@NiO–TiO_2_ nanoparticles could have beneficial applications in environmental remediation and biological science.

## Abstract 2

A synergistically engineered Ag@NiO–TiO_2_ ternary nanocomposite was synthesized using hydrothermal method, and its photocatalytic performance was evaluated for the degradation of organic dye and antibiotic contaminants.

## Graphical abstract

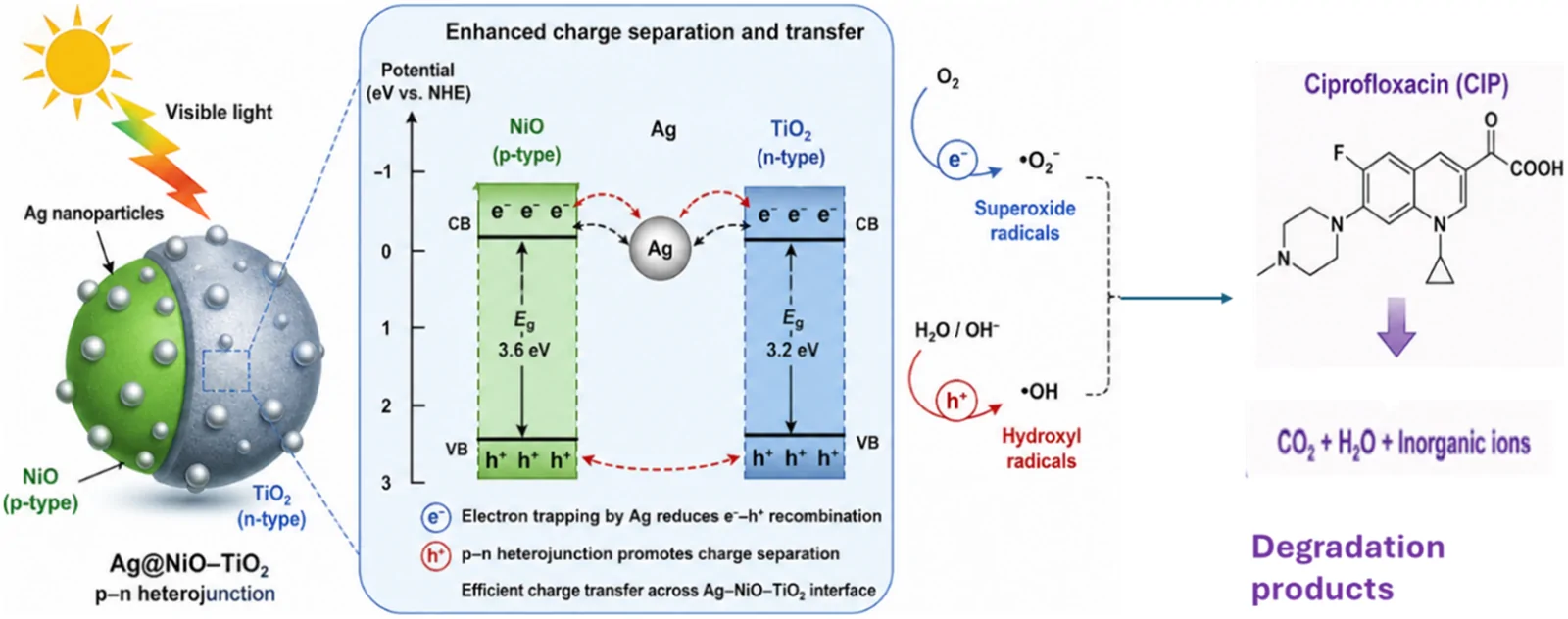

## 1. Introduction

Organic pollutants, such as methylene blue and crystal violet dyes, collected from industries contain complex aromatic compounds that are non-biodegradable, environmentally toxic and carcinogenic.^1^ It is highly recommended to remove these colored compounds from sewage wastewater before it enters natural habitats due to their toxicity, bioactivation properties, complex chemical nature and cancerous proclivity.^2,3^ Methylene blue is applied for different purposes such as dyeing cotton, silk, wool, *etc.* Similarly, crystal violet is a triphenylmethane dye that is used in the textile processing industry as well as a biological stain. These dyes are associated with harmful side effects on human health, and hence, it is essential to remove these dyes from wastewater on a priority basis.^4–6^ Similarly, based on their worldwide sales and therapeutic versatility, fluoroquinolone antibiotics have been one of the most widely used medications in veterinary medicine since the 1960s.^7^ High quantities of ciprofloxacin (CIP), a significant member of this group, and its products have been detected in water sources in the range of ng L^−1^ to µg L^−1^ since it is widely employed to treat illnesses caused by bacterial infections. When this antibiotic medication is present in an aqueous medium, it can become more stable, resulting in harmful effects like causing cancer. Consequently, using a practical and sustainable approach to address this issue is essential.^8^ Two of the global concerns for human health are bacterial and fungal infections.^9,10^ The World Health Organization published a report in 2014 that in the face of microbial illness, if the excessive use of antibiotics continues at the current rate, the resulting rise in antimicrobial resistance may severely limit the effectiveness of available treatments for bacterial infections.^11^ It is assessed that human mortality rate can increase to 12 million people in the next 30 years as compared with 700 000 people due to such drug-resistant bacterial infections.^12^ The World Health Organization has identified fungal infections and antifungal resistance as growing major public health concerns that require urgent research on therapeutic agents.^13^ Therefore, it is essential to develop novel chemically altered compounds to respond to these multidrug-resistant bacterial and fungal infections.^14^

Semiconductor-based oxidative photocatalysis technology has shown great progress and attracted attention in the degradation of organic dyes and antibiotics to deactivate bacteria and viruses.^15^ Semiconductors such as zinc oxide, titanium oxide, manganese oxide, cadmium sulphide and g-C_3_N_4_ are well known for their photocatalytic and antimicrobial activities. These photocatalysts are highly efficient, inexpensive, less toxic, easily available, environmentally friendly, and highly stable in a wide range of pH with high oxidizing photo-generated hole properties.^16–19^ When these materials are used as photocatalysts, they suffer from several drawbacks, such as wide band gap range, incomplete mineralization and fast recombination of electrons (e^−^) and holes (h^+^), which decreases their photocatalytic activities.^20^ Therefore, to increase the efficiency of these materials, several metal oxide nanomaterials, doped metal oxides and metal oxide nanocomposites were prepared and investigated.^20–22^ The TiO_2_ photocatalytic activity is improved by doping it with different metals such as Ni, Ag, Pd and Ni nanoparticles. More specifically, the ability of Ag and Au to produce Schottky heterostructures with TiO_2_ has led to their widespread use. However, compared to Au nanoparticles, Ag nanoparticles are more cost-effective and have a larger work function. Additionally, Ag nanoparticle doping increases the efficiency of electron injection into TiO_2_ because the Ag nanoparticles' SPR induces charge carriers. As a result, the surface plasmon resonance excitations of Ag nanoparticles in the shorter wavelength area of the visible light spectrum (about 380–450 nm) would increase the TiO_2_ photocatalytic activity.^23,24^ However, when TiO_2_ and p-type NiO are combined, a p–n heterojunction is created that reduces photogenerated charge carrier recombination and increases charge separation *via* an internal electric field.^25^ Ag nanoparticles doping further enhances the properties of systems by extending visible-light absorption through localized SPR, creating Schottky barriers that trap electrons, and gives intrinsic antibacterial activity.^26^

The synergistic effect of Ag, NiO, and TiO_2_ was confirmed by a recent work on ternary Ag@NiO–TiO_2_ heterojunctions, which showed improved UV-visible light degradation of pollutants and pharmaceutical wastes compared with TiO_2_, Ag/TiO_2_, and NiO/TiO_2_.^24^ Ag@NiO–TiO_2_ nanomaterials are potential multifunctional materials for photodegradation and antimicrobial applications due to the incorporation of TiO_2_ with the p-type semiconducting material of NiO and the plasmonic/antimicrobial activity of Ag nanoparticles. Ag@NiO–TiO_2_ is therefore selected as a new photocatalytic-antimicrobial material due to its superior visible-light activation, reduced charge recombination, increased formation of reactive oxygen species, better degradation efficiency, and significant potential for bacterial inhibition. The results might be helpful in the development of advanced nanomaterials for environmental disinfection, dye and pharmaceutical contaminant degradation, and wastewater treatment.

The present work reports the development of Ag@NiO–TiO_2_ ternary nanomaterials as a dual-functional platform for both the photocatalytic removal of dye/pharmaceutical contaminants and antibacterial activities. The core objective of this work is to synthesize and characterize Ag@NiO–TiO_2_ nanomaterials and evaluate their efficiency for the UV-visible light-assisted removal of methylene blue, crystal violet, and ciprofloxacin, along with their antibacterial performance against selected pathogenic bacterial and fungal strains. This work offers a versatile and sustainable nanomaterial-based method for environmental disinfection and wastewater treatment.

## 2. Experimental

### 2.1. Materials

Titanium isopropoxide (97%), AgNO_3_ and nickel nitrate hexahydrate (Ni(NO_3_)_2_·6H_2_O) were procured from SRLChem USA. Sodium borohydride (NaBH_4_, Fisher Scientific), ethanol (EtOH, Alfa Aesar Chemicals), methylene blue and crystal violet dyes (Sigma-Aldrich, USA), and ciprofloxacin (Pharmaceutical grade, USA) were used as received with any further purification.

### 2.2. Synthetic procedure for TiO_2_, NiO–TiO_2_, and Ag@NiO–TiO_2_ nanocomposites

TiO_2_ nanoparticles were synthesized by dissolving 10 mL of TTIP into 40 mL of isopropanol to obtain solution A. Then, 5 mL of acetic acid (solution B) was added into 100 mL of deionized water in a separate beaker. Under constant stirring solution A was added dropwise into solution B. The solution was put for aging for 12 hours and then dried at 80 °C. TiO_2_ nanoparticles were obtained by calcining at 500 °C for 6 h. The collected solid yield was 2.3 g, corresponding to 88% yield.^27^

The synthesis of ternary NiO–TiO_2_ heterojunction nanocomposites was carried out through hydrothermal methods. NiO–TiO_2_ nanocomposites were prepared by mixing 2 : 1 ratio of TiO_2_ (2 g) and NiO (1 g) in ultra-pure water, and then this solution was transferred to an autoclave. At 200 °C temperature, a standard calculated quantity of NiO and TiO_2_ dispersed in 100 mL of deionized water were subjected to 12 h reaction in the autoclave. The mixture was then filtered, washed several times with deionized water and methanol, and dried at 70 °C for 12 h to obtain the NiO–TiO_2_ nanocomposite. The collected amount of the NiO–TiO_2_ composite was 2.9 g, equal to 96% yield.^28^

To synthesize the Ag@NiO–TiO_2_ photocatalyst, 1.0 g of TiO_2_ powder was sonicated in 60 mL of deionized water for 20–30 minutes, and then, the mixture was agitated magnetically for 30 minutes to obtain a homogeneous suspension. After dissolving the required quantities of 0.5 g of NiO and 0.03 g AgNO_3_ separately in deionized water, both precursor solutions were added dropwise into the TiO_2_ suspension under vigorous stirring. The pH was adjusted to about 9–10 using diluted NaOH and stirring was continued at 60–80 °C for two hours. Then, the solution was transferred to an autoclave and the reaction was carried out for 12 h at 180 °C. The suspension was centrifuged, repeatedly cleaned with deionized water until the Ph is neutral, washed several times with methanol, and dried overnight at 80 °C.^29^ The collected amount of the solid was 1.32 g, which was approximately 86% yield. The synthetic method is shown in Scheme 1.

**Scheme 1:**
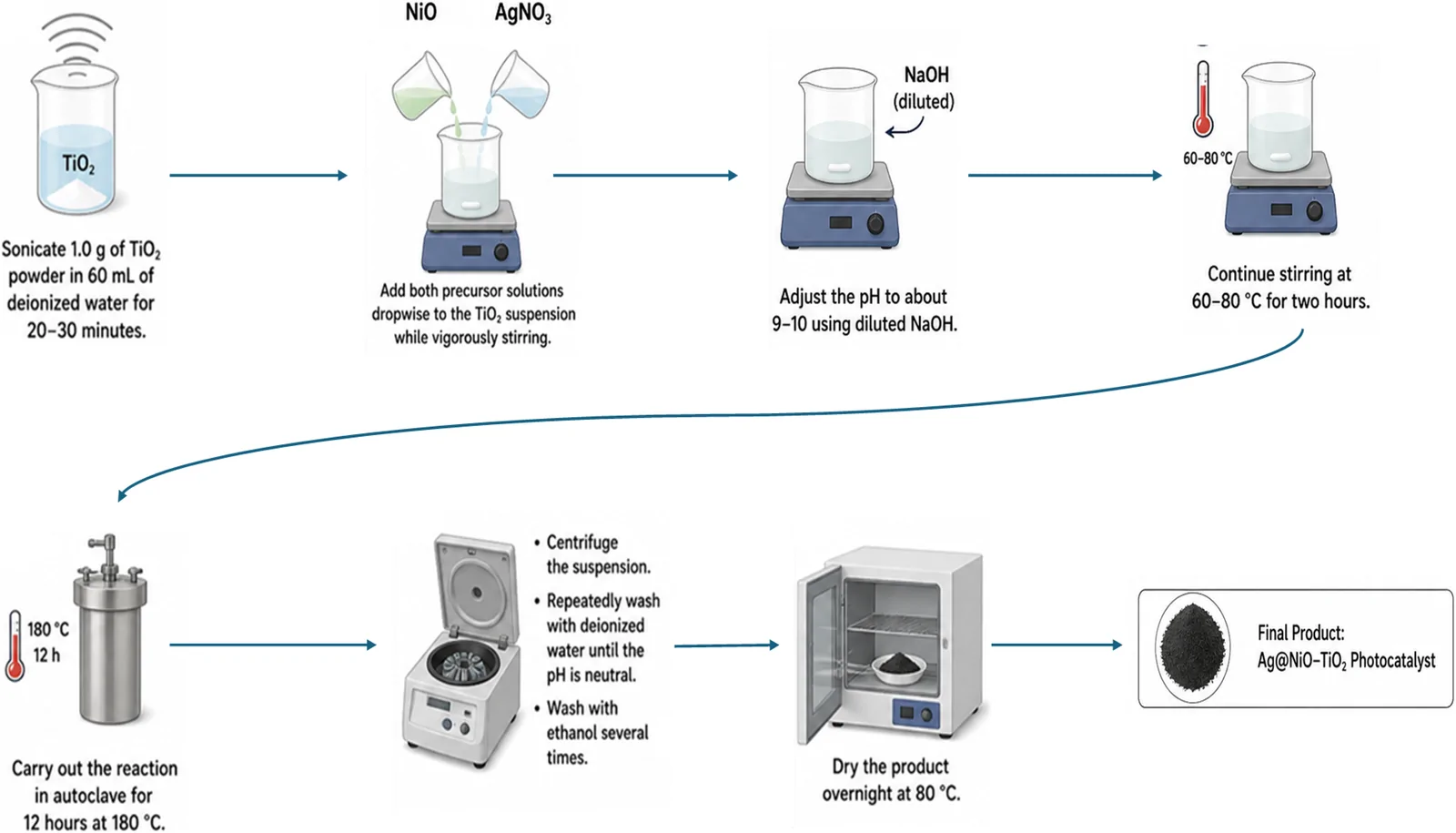
Schematic of the synthesis procedure of the Ag@NiO–TiO_2_ ternary heterojunction.

### 2.3. Material characterization

The present work studied the structure, optical properties, morphology, elemental composition, and particular peaks of TiO_2_, NiO–TiO_2_, and Ag@NiO/TiO_2_ nanocomposites using FT-IR spectroscopy, X-ray diffraction (XRD), UV-vis spectroscopy, Raman spectroscopy, PL, SEM-EDX, TEM and TGA. A Jasco Fourier transform infrared (FTIR-4700) spectroscopy instrument was used to examine the presence of bonds between the composite materials. Powder X-ray diffraction (XRD) using a Bruker Advance D8 diffractometer with Cu Kα radiation of *λ* = 0.154 nm was performed to analyze the crystalline structure of the synthesized titanium oxide nanoparticles and their composites. The UV-vis spectroscopy results of the samples were obtained using an Analytik Jena SPECORD 200 Plus UV-vis spectrophotometer. Raman measurements (*λ*_ex_ of 785 nm, 200–2500 cm^−1^) were carried out using a Renishaw Ramascope. The morphological features and EDS were examined using an FESEM Hitachi SU8020. Similarly, transmission electron microscopy (Thermo Fisher Scientific (TFS) Talos system operating at 200 kV) was used to examine the shape, size and structure of the catalyst samples. The thermal stability was investigated using a TGA Q500 model.

### 2.4. Experimental procedure

MB and CV dyes and ciprofloxacin antibiotic in an aqueous solution were used to assess the efficiency of the catalyst to degrade the pollutants. All photodegradation studies were conducted using a 400 W halogen lamp (metal halide) with a UV cut filter coating (*λ* > 400 nm, with *λ*_max_ = 546 nm). Using 20 mg loading of photocatalyst suspended in 100 mL of MB, CV and CIP (10 mg L^−1^) solution in a 150 mL glass reactor, the light source intensity was measured using a light meter at 15 cm between the lamp and the cup. It was approximately 170 W m^−2^. The solution was constantly stirred in the dark for fifteen minutes before irradiation to balance the absorption and desorption processes between the photocatalyst and the MB, CV, and CIP solution. To assess the degradation of the MB and CV dye and CIP, absorbances were measured at specified intervals. The aqueous solution was exposed to radiation in the control experiment using a photocatalyst. After the catalyst was extracted from the liquid using a 0.45 µm PTFE syringe filter, the MB concentration was determined by UV-vis spectrometry. Moreover, the degradation efficiency of the catalysts can be calculated using eqn (1) as follows:1
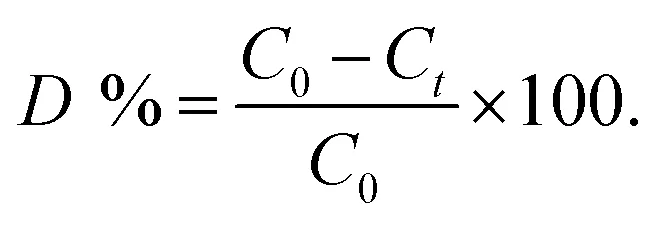


### 2.5. Antimicrobial activities

#### 2.5.1. Antibacterial activity

The previously reported method was used to examine the susceptibility of the compound to various bacterial species. Three Gram-negative organisms were examined: *Enterobacter aerogenes*, *Salmonella typhimurium*, and *Bordetella bronchiseptica*. The strains were cultivated in a nutrient broth medium and incubated at 37 °C for a whole day. The turbidity was adjusted to 10^4^ CFU mL^−1^ using sterilized deionized water and compared to McFarland 0.5 BaSO_4_ turbidity standards. After that, 100 µL of the updated inoculum was swabbed onto Petri dishes with 20 mL of nutritional agar. Sterile filter paper discs (6 mm) were injected with test samples (5 µL of 20 mg per mL DMSO; 100 µg per disc) and put on a seeded nutrient agar plate. Positive and negative controls were DMSO-impregnated discs and cefixime-USP at a dose of 20 µg per disc, respectively.^30^

#### 2.5.2. Antifungal activity

Antifungal activity was assessed using a standard procedure reported previously.^31^ SDA was used to cultivate the fungal strains *Aspergillus fumigatus*, *Fusarium solani*, and *A. niger* acquired from the Fungal Culture Bank of Pakistan. The spores were collected in a 0.02% Tween 20 solution, and their turbidity was corrected using the McFarland 0.5 turbidity standard prior to the sensitivity assessment. On plates with 25 mL of sterilized SDA, 100 µL of each extracted spore was swabbed. On seeded SDA plates, filter paper discs containing 5 µL of the test sample (20 mg per mL DMSO; 100 µg per disc), standard antifungal terbinafine (50 µg per disc), and DMSO were added. For 24 to 48 hours, the plates were incubated at 28 °C. A Vernier caliper was then used to measure the clear zones of inhibition surrounding the discs.

Zone of inhibition (ZOI) and minimum inhibitory concentration (MIC) measurements of the antibacterial tests were carried out in the presence of visible light. Consequently, the material's performance under light-activated conditions, where photocatalytically produced reactive oxygen species (ROS) can considerably contribute to bacterial inactivation, is reflected in the reported antimicrobial activity.^32^

## 3. Results and discussion

### 3.1. Characterization

#### 3.1.1. FT-IR analysis

Fig. 1a displays the FT-IR spectra for pure TiO_2_, NiO–TiO_2_ and Ag@NiO–TiO_2_ nanocomposites recorded in the 400–4000 cm^−1^ range. The existence of O–H stretching and bending modes of vibrations resulting from water absorption is responsible for the strong wide band at around 3200–3500 cm^−1^ in all the synthesized samples. A characteristic vibration mode of TiO_2_ is shown by the appearance of a wide absorption band at 552 cm^−1^.^33^ For the NiO–TiO_2_ nanocomposite sample, broad peaks of TiO_2_ and NiO appear at about 688 cm^−1^ and 470 cm^−1^, respectively, as a shift was observed in the peak due to the presence of NiO, which is commonly reported in the literature.^34^ For the Ag@NiO–TiO_2_ nanocomposite sample, a small change appears in the peak position, and no clear new peak is observed for Ag itself, indicating that the ternary heterojunction was successfully formed. The broad absorption band at 3395 cm^−1^ is attributed to the O–H stretching vibration of surface hydroxyl groups and physically bound water molecules on the surface of the Ag@NiO–TiO_2_ compound.^24^

**Fig. 1:**
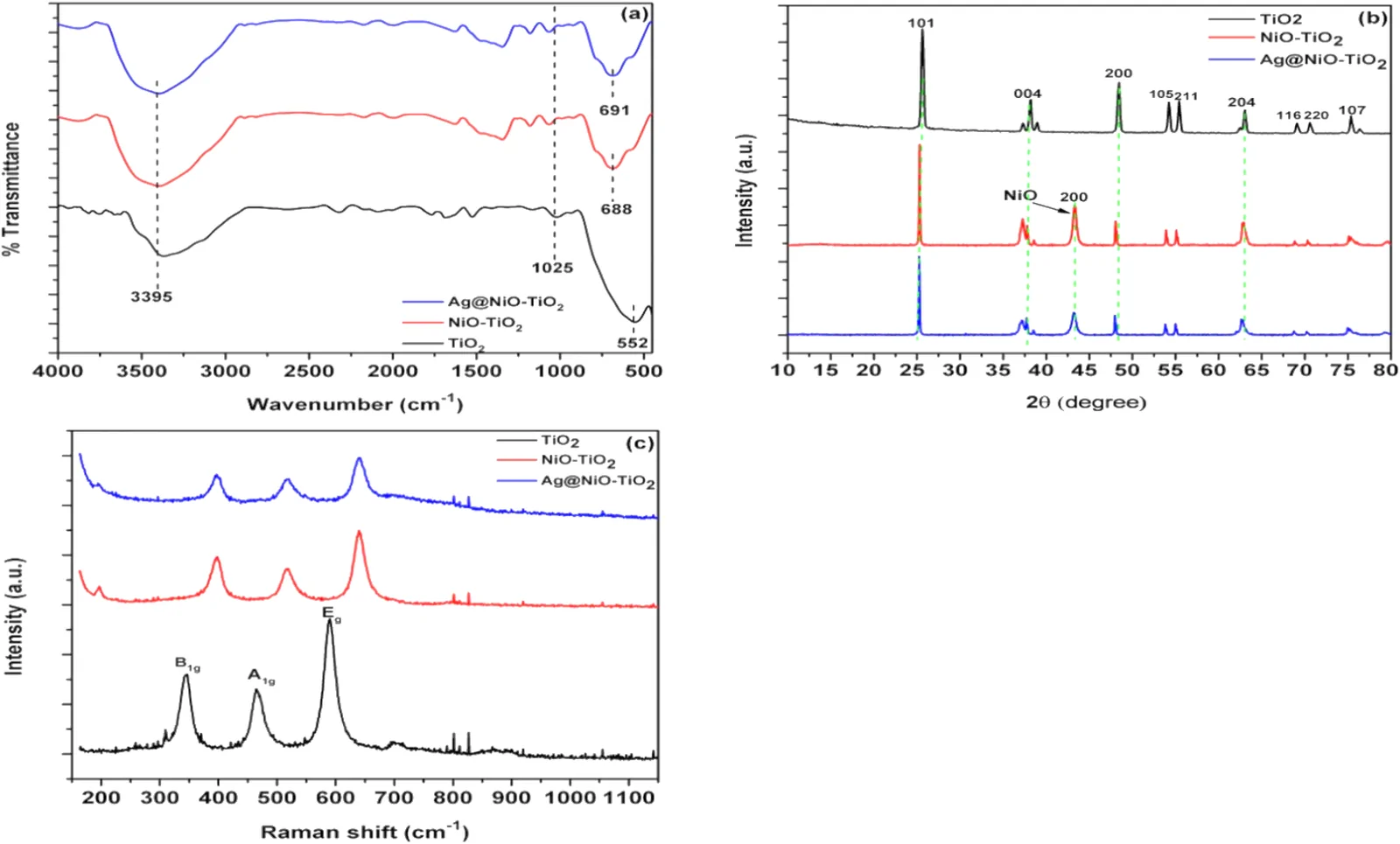
(a) FT-IR spectra of the TiO_2_, NiO–TiO_2_ and Ag@NiO–TiO_2_ nanoparticles. (b) XRD patterns of the TiO_2_, NiO–TiO_2_ and Ag@NiO–TiO_2_ nanoparticles. (c) Raman spectra of the TiO_2_, NiO–TiO_2_, and Ag@NiO–TiO_2_ nanoparticles.

#### 3.1.2. XRD analysis

XRD analysis showed that after adding NiO and subsequently Ag, as shown in Fig. 1b, the three patterns maintained almost the same primary peak positions, which often indicates that the basic TiO_2_ anatase crystal structure is still retained. The dominating anatase TiO_2_ (101) reflection is consistent with the strongest peak at about 25°, and the other recurrent peaks likewise correspond to frequent anatase sites.^35^ Metallic Ag diffraction peaks appear near 38°, 44°, 64°, and 77°, while the peaks around 37° and 43° are typically associated with cubic NiO. The NiO–TiO_2_ and Ag@NiO–TiO_2_ traces show these additional weak peaks and intensity changes, suggesting the successful loading of secondary phases or surface modification rather than a complete phase transformation of TiO_2_.^36^ Anatase TiO_2_ continues to be the predominant crystalline phase in all samples, according to the XRD patterns. Furthermore, using Cu Kα radiation with a wavelength of 1.5406 Å and the Debye Scherrer equation (crystallite size (nm) = 0.9 *λ*/*B* cos *θ*) and Williamson–Hall (W–H) analysis was used to determine the sample crystallite size and lattice strain from the XRD data. The major anatase TiO_2_ diffraction peaks at around 25.28°, 37.78°, 48.02°, 53.87°, and 55.04° were used for analysis. The crystallite size was determined from these peaks, according to the Debye Scherrer equation, with an average of roughly 60.2 ± 6.4 nm. Using the formula, *β* cos *θ* = 4*ε* sin *θ* + *Kλ*/*D*, Williamson–Hall analysis gave an estimated crystallite size of about 59.1 nm and a lattice strain of around −1.54 × 10^−5^, correspondingly about −0.0018%. The negative sign suggests a tiny compressive strain, and the very small magnitude of the strain showed that the lattice deformation in the sample is almost insignificant.

#### 3.1.3. Raman analysis

The crystalline TiO_2_ phase was confirmed by the black TiO_2_ spectrum, which displayed distinctive Raman peaks at 350–400, 460, and 590–650 cm^−1^, as shown in Fig. 1c. The red NiO–TiO_2_ spectrum retained identical peak positions when NiO was added, but the peaks grew larger and slightly displaced, indicating potential structural distortion and interaction between NiO and TiO_2_. The Raman peaks are still visible in the blue Ag@NiO–TiO_2_ spectrum, but they look larger and less strong than those for pure TiO_2_. This suggests that Ag insertion changes the surface structure and promotes defect formation or charge interaction. Overall, the spectra showed that TiO_2_ is still the primary phase in all samples, but the addition of NiO and Ag affects the composite material crystallinity, surface defects, and electrical interaction. These Raman spectra variations are frequently linked to strain caused by dopants and heterojunctions, nanoscale effects, and modified charge-transfer behavior in metal oxide nanocomposites. The successful synthesis of NiO–TiO_2_ and Ag@NiO–TiO_2_ heterostructures, where NiO and Ag affect the surface structure and electronic interaction of TiO_2_, is thus supported by the Raman study.^37,38^

The vibrational modes of TiO_2_ are primarily responsible for the Raman peaks seen in the TiO_2_, NiO–TiO_2_, and Ag@NiO–TiO_2_ spectra. The crystalline TiO_2_ structure is confirmed by the *E*_g_ mode, which shows stretching vibrations of the Ti–O bond. Ti–O–Ti bending vibrations are associated with the B_1g_ mode, whereas symmetric stretching vibrations of the Ti–O framework are associated with the A_1g_ mode. These modes existence in every sample confirms the TiO_2_ phase retention following NiO and Ag incorporation. Slight variations in peak intensity, broadening or shifting, however point to defect development, lattice distortion, and interactions between Ag, NiO, and TiO_2_ in the nanocomposite structure.^39^

#### 3.1.4. SEM analysis

The SEM image of pure TiO_2_ (Fig. 2a and b) shows a porous network formed by densely agglomerated, roughly spherical nanoparticles. Sol–gel-derived TiO_2_ exhibits such aggregated nanocrystalline TiO_2_ domains, which offer a high surface area advantageous for photocatalytic applications.^40^ The NiO–TiO_2_ nanocomposite (Fig. 2c and d) exhibits a more compact surface, featuring sub-nanometer aggregates made up of finer nanograins. The difference in texture when compared to pure TiO_2_ suggests that NiO particles/shells are closely deposited on the TiO_2_ matrix. This aligns with prior findings on NiO–TiO_2_ heterostructures, where NiO incorporation enhances surface roughness and creates a p–n junction with TiO_2_.^41^ The high-magnification SEM image of Ag@TiO_2_–NiO (Fig. 2e and f) shows fine nanoparticles (8–16 nm) that are uniformly distributed on the NiO–TiO_2_ aggregates, as indicated by the size markers. Ag nanoparticles that are anchored on the NiO–TiO_2_ surface are the origin of these nanodots. Similar sizes and distributions of Ag nanoparticles on TiO_2_ are observed in Ag–TiO_2_ and Ag@NiO–TiO_2_ systems, where the Ag NPs create Schottky contacts and enhance visible-light absorption through plasmonic effects.^42^

**Fig. 2:**
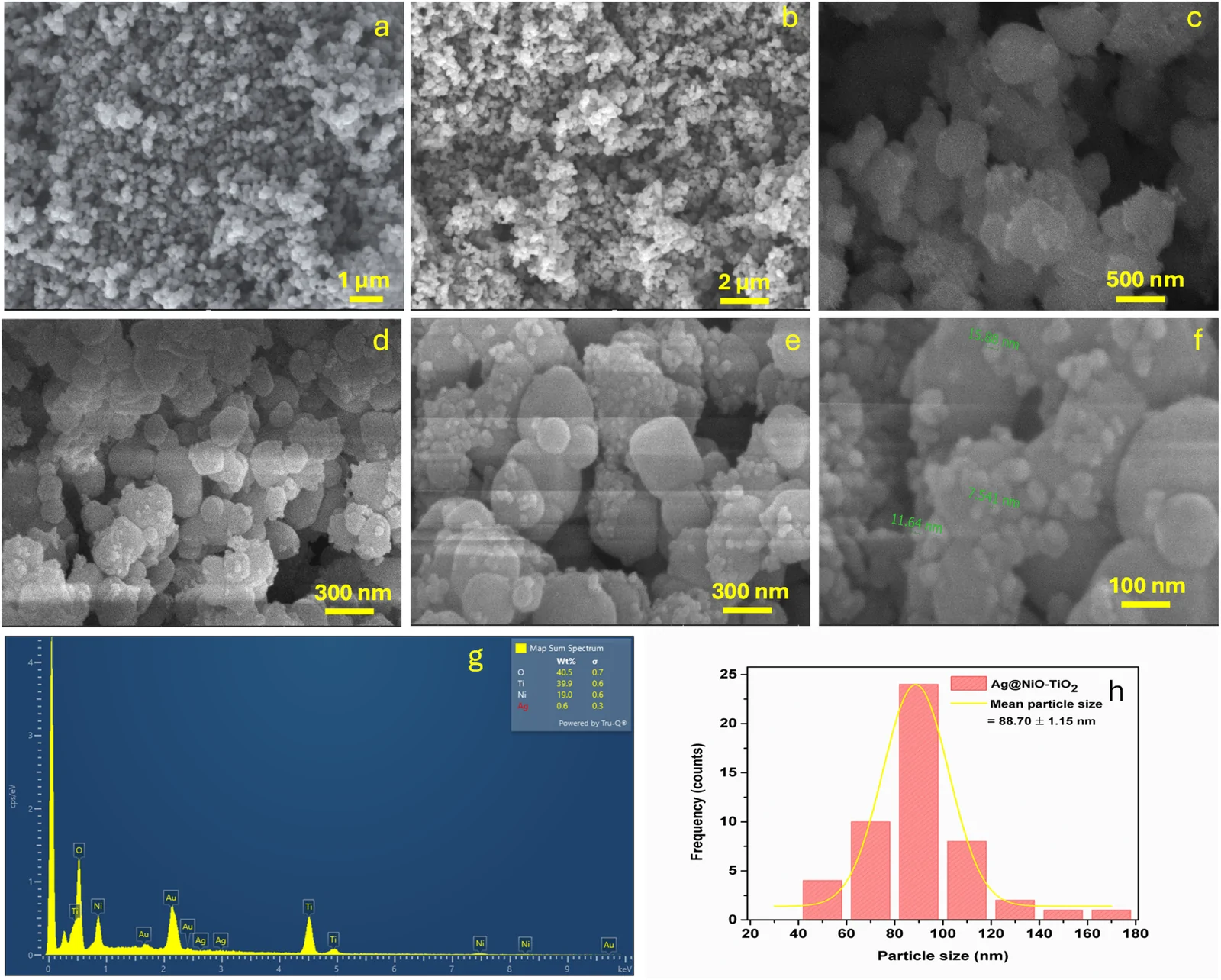
SEM images of TiO_2_ (a and b), NiO–TiO_2_ (c and d), and Ag@NiO–TiO_2_ (e and f) under different magnifications. EDS spectrum of the Ag@NiO–TiO_2_ nanoparticles (g) and particle size distribution histogram of Ag@NiO–TiO_2_ nanoparticles (h).

#### 3.1.5. EDS analysis

The successful synthesis of the Ag@NiO–TiO_2_ nanocomposite is shown by this EDX spectrum in Fig. 2g; which confirms the presence of O, Ti, Ni, and Ag in the sample. TiO_2_ is confirmed as the predominant phase by the greatest elemental contributions from titanium (39.9 wt%) and oxygen (40.5 wt%). The integration of NiO into the TiO_2_ matrix is supported by the presence of nickel (19.0 weight percent). The detection of a tiny quantity of silver (0.6 weight percent) indicates the presence of Ag on the composite surface, though at a low concentration. The elemental mapping showed the Au peaks, which are caused by gold plating or the sample holder used during EDX analysis. Overall, the spectrum confirms that the Ag-modified NiO–TiO_2_ material has the predicted element makeup. SEM-derived particle size distribution histogram of Ag@NiO–TiO_2_ NPs in Fig. 2h revealed that the particles are mostly found in the 60–120 nm range, with a maximum frequency of 80–100 nm. The average particle size, according to the Gaussian fitting, is around 88.70 nm. Particle aggregation may be the cause of the observed modest positive tilt toward bigger particle sizes.

#### 3.1.6. TEM analysis

TiO_2_ nanoparticles with primarily spherical to irregular forms are shown in Fig. 3a and b. Due to their high surface energy, oxide nanoparticles are frequently large and seem agglomerated. The overlapping particles of varying thicknesses are observed in images having contrast variation. The smaller particles of the NiO–TiO_2_ nanocomposite are dispersed around or on the surface of larger TiO_2_ particles following NiO inclusion, as shown in Fig. 3c and d. Fine NiO–TiO_2_ nanoparticles are seen to cluster together as the shape gets more altered. This shows that NiO and TiO_2_ have successfully coupled to create a composite structure. Similarly, the Ag@NiO–TiO_2_ nanocomposite is shown in Fig. 3e and f. The larger oxide particles are decorated with small dark nanoparticles, which might be Ag and/or NiO nanoparticles on TiO_2_ surfaces. Strong interactions between Ag, NiO, and TiO_2_ and the successful Ag loading are shown by the particles' increased agglomeration and strong interaction.^43^ Therefore, the synthesis of NiO–TiO_2_ and Ag@NiO–TiO_2_ nanocomposites was confirmed by the TEM images showing distinct morphological changes. The effective fabrication of heterostructure nanocomposites with close contact between TiO_2_, NiO, and Ag is supported by these findings.

**Fig. 3:**
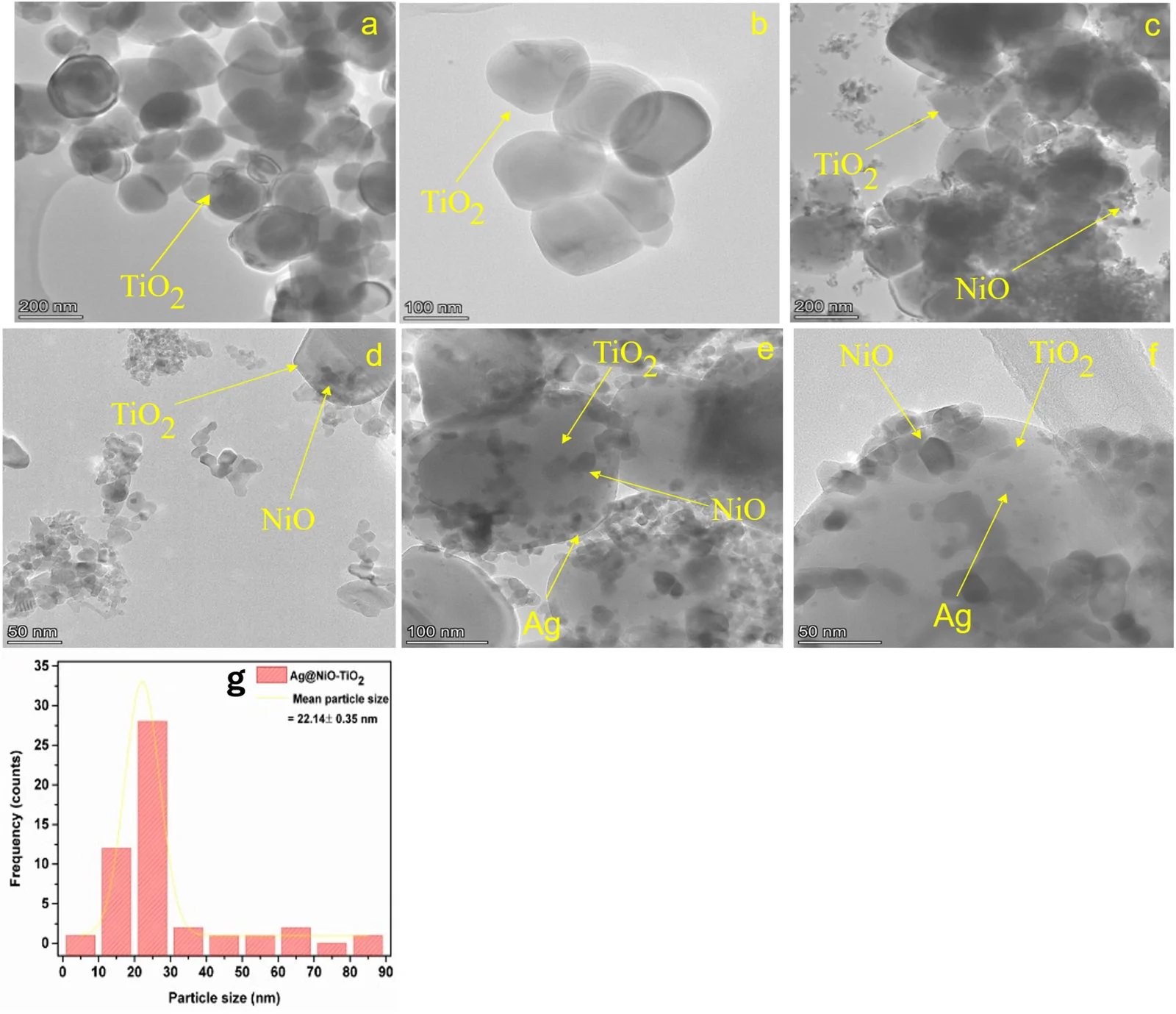
Standard TEM images of TiO_2_ (a and b), NiO–TiO_2_ (c and d) and Ag@NiO–TiO_2_ (e and f) nanocomposites under different magnifications.and particle size distribution histogram from TEM of Ag@NiO–TiO_2_ nanoparticles (g).

#### 3.1.7. UV-vis analysis

Using UV-visible (UV-vis) spectroscopy, the energy band gap of the Ag@NiO–TiO_2_ photocatalyst was examined and compared with those of TiO_2_ and NiO–TiO_2_ composites, as seen in Fig. 4a and b. The absorbance values of the samples were measured in the 350–900 nm range. The red shift at 450 nm in Fig. 4a indicates that adding Ag and NiO nanoparticles to TiO_2_ greatly enhanced the composite absorption. Hence, the Ag nanoparticles impact localized surface plasmon resonance (LSPR), and the shift of the peak to a longer wavelength value (redshift) will result in a decrease in the energy band gap of Ag@NiO–TiO_2_. In addition, the color shift to brown following the synthesis of Ag@NiO–TiO_2_ in contrast to the white rutile TiO_2_ is another indication, which promotes the absorption spectrum of visible light. The Tauc plot of TiO_2_ is displayed alongside NiO–TiO_2_ and Ag@NiO–TiO_2_ composites in Fig. 4b. The plot showed that all compounds have band gap energies for TiO_2_, NiO–TiO_2_ and Ag@NiO–TiO_2_ nanoparticles as 3.37, 3.35 and 3.27, respectively, as determined using the Tauc plot method. These findings show that the band gap was lowered by 0.10 eV when Ag and NiO were added to TiO_2_.

**Fig. 4:**
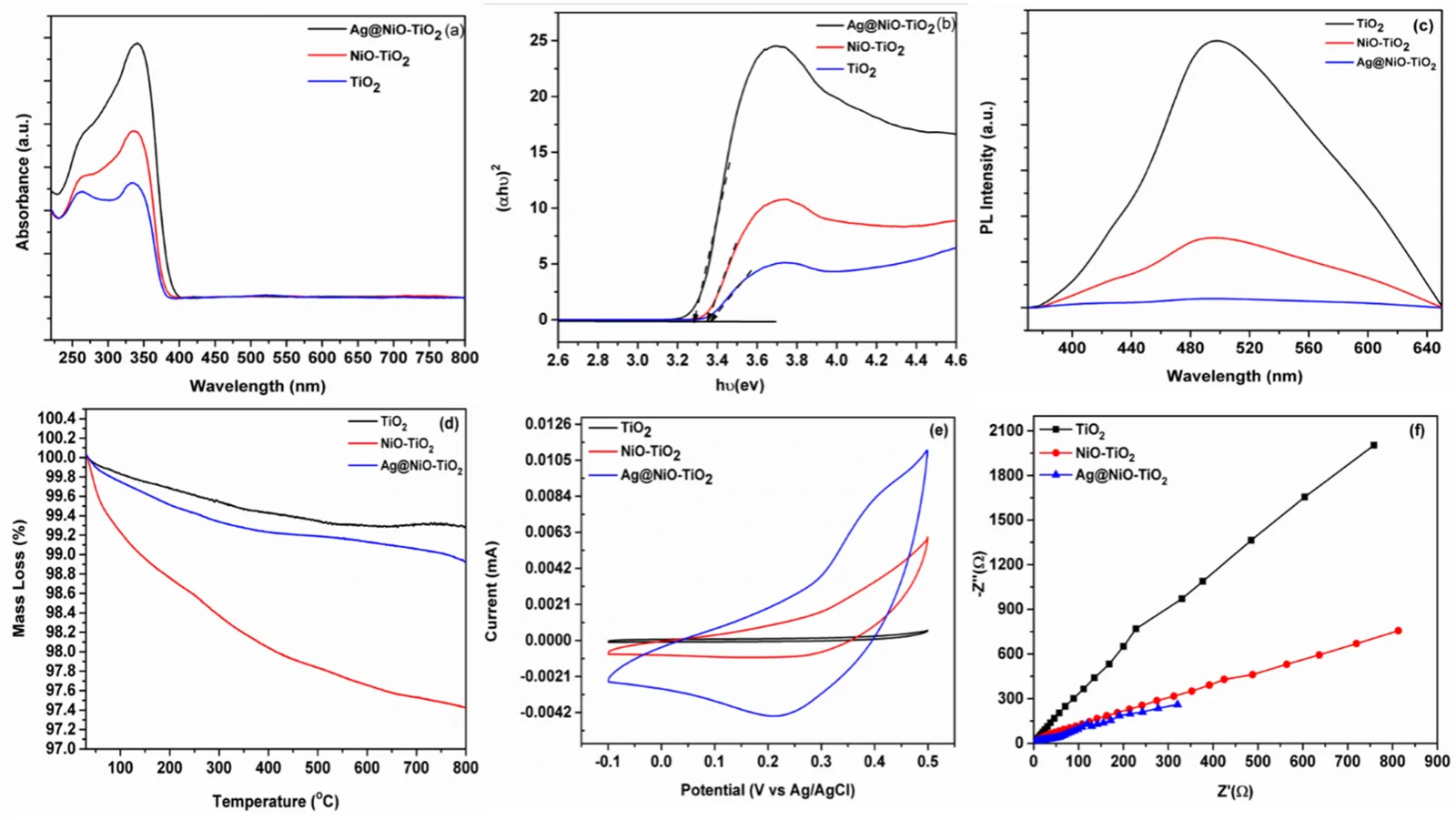
(a) UV-vis spectral comparison of titanium oxide (TiO_2_), titania–nickel oxide (NiO–TiO_2_), and the ternary silver-doped titania–nickel oxide nanocomposite (Ag@NiO–TiO_2_). (b) Band gap energy of the nanoparticles using the Tauc plot of the corresponding results. (c) PL spectra of TiO_2_, NiO–TiO_2_ and the Ag@NiO–TiO_2_ nanocomposite. (d) TGA graphs of TiO_2_, NiO–TiO_2_, and Ag@NiO–TiO_2_ in the 25–800 °C temperature range. Electrochemical characterization of TiO_2_, NiO–TiO_2_ and the Ag@NiO–TiO_2_ nanocomposite: (e) cyclic voltammetry and (f) EIS Nyquist plots.

#### 3.1.8. Photoluminescence spectroscopy analysis

The photoluminescence spectra provide insights into the recombination behavior of photogenerated charge carriers in the photocatalysts, as displayed in Fig. 4c. Pure TiO_2_ exhibits the highest PL intensity, which indicates rapid recombination of photoexcited electrons and holes, limiting its photocatalytic activity. After coupling TiO_2_ with NiO, the PL intensity decreases, suggesting that the formation of a NiO–TiO_2_ heterojunction promotes interfacial charge transfer and improves electron–hole separation.^28^ The Ag@NiO–TiO_2_ composite shows the lowest PL emission intensity, confirming the most efficient suppression of charge-carrier recombination. Ag nanoparticles function as conductive mediators and electron traps, which promote charge separation and electron movement responsible for this improvement, whereas NiO facilitates the creation of heterojunctions with TiO_2_ on its surface. As a result, the lower PL intensity of Ag@NiO–TiO_2_ suggests improved charge-transfer efficiency, a longer carrier lifetime, and increased availability of reactive species such ˙OH and ˙O_2_^−^, which improves the photocatalytic degradation performance.^44^

#### 3.1.9. TGA

The thermal stability of TiO_2_, NiO–TiO_2_, and Ag@NiO–TiO_2_ from room temperature to 800 °C is shown in Fig. 4d. The materials are thermally stable since all three samples exhibit very little mass loss. With a mass loss of about 99.3%, pure TiO_2_ exhibits the least amount of mass loss, indicating strong thermal stability. Ag@NiO–TiO_2_ exhibits a little greater mass loss, close to 98.9%, indicating some surface alteration and adsorbed species removal. The removal of physically adsorbed water, surface hydroxyl groups, or leftover precursor species from the composite surface may be the cause of NiO–TiO_2_ largest mass loss, which ends at about 97.4%. While slow weight loss at higher temperatures may be linked to structural/surface changes or the breakdown of residual organics, low-temperature mass loss in TiO_2_-based nanomaterials is often related to adsorbed water and surface hydroxyl groups. TGA is frequently used to assess how the weight of nanoparticles changes and thermal stability is affected as the temperatures increases. The TiO_2_, NiO–TiO_2_, and Ag@NiO–TiO_2_ TGA curves show a slow mass loss as the temperature rises, indicating that all samples have high thermal stability up to 800 °C.^45^

#### 3.1.10. Electrochemical properties by CV

In comparison to TiO_2_ and NiO–TiO_2_, the graph reveals that the Ag@NiO–TiO_2_ nanocomposite exhibits improved charge-transfer behavior, which is advantageous for both photocatalytic and antimicrobial applications. Improved electrochemical activity and higher separation/mobility of photogenerated electron–hole pairs are suggested by the bigger current response and wider redox region in the cyclic voltammetry curve.^46^ Ag inclusion increases the conductivity and decreases the charge carrier recombination, as seen by the comparison plot on the right in Fig. 4e, which also displays a greater response for the Ag-modified nanocomposite. These characteristics increase the production of ROS when exposed to radiation, which improves the breakdown of contaminants like dyes and antibiotics. Stronger antimicrobial action can also arise from high generation of reactive species, which can harm bacterial cell walls, proteins, and DNA.

The charge-transfer activity of the prepared photocatalysts is shown in Fig. 4f. The curve showed reduced impedance/charge-transfer resistance in electrochemical impedance spectroscopy, indicating improved electron–hole pair separation and quicker photogenerated electron mobility. The Ag@NiO–TiO_2_ nanocomposite exhibits the best EIS response when compared to NiO–TiO_2_ and TiO_2_, indicating that Ag insertion enhances interfacial charge transfer and electrical conductivity. When exposed to light, this decreased resistance aids in preventing electron–hole recombination. Therefore, there are more electrons and holes available to produce reactive oxygen species such OH˙ and O_2_˙^−^ radicals, which improve the photocatalytic destruction of contaminants.^47^ The improved photocatalytic activity of Ag@NiO–TiO_2_ is therefore supported by the EIS findings.

### 3.2. Photocatalytic activity

#### 3.2.1. MB dye photodegradation

The time-resolved UV-vis spectra of MB reveal a gradual reduction in the characteristic band at 665 nm for all catalysts, confirming photocatalytic degradation. Due to its wide band gap and rapid charge recombination, TiO_2_ demonstrates only moderate MB removal after 60 minutes, as presented in Fig. 5a. It showed 51% removal of MB dye after one hour. The decrease in MB absorbance in TiO_2_–NiO occurs at a significantly accelerated rate, which is attributed to the p–n heterojunction that enhances charge separation and the use of visible light, and the removal of the dye reached 76% after 60 minutes, as presented in Fig. 5b. The highest activity is shown by the Ag@NiO–TiO_2_ nanocomposite that approximately removes 88% of MB in 60 min, as presented in Fig. 5c. This improvement is due to the combined effects of the NiO–TiO_2_ heterojunction and plasmonic Ag, which enhance light absorption and reduce electron–hole recombination.^48^

**Fig. 5:**
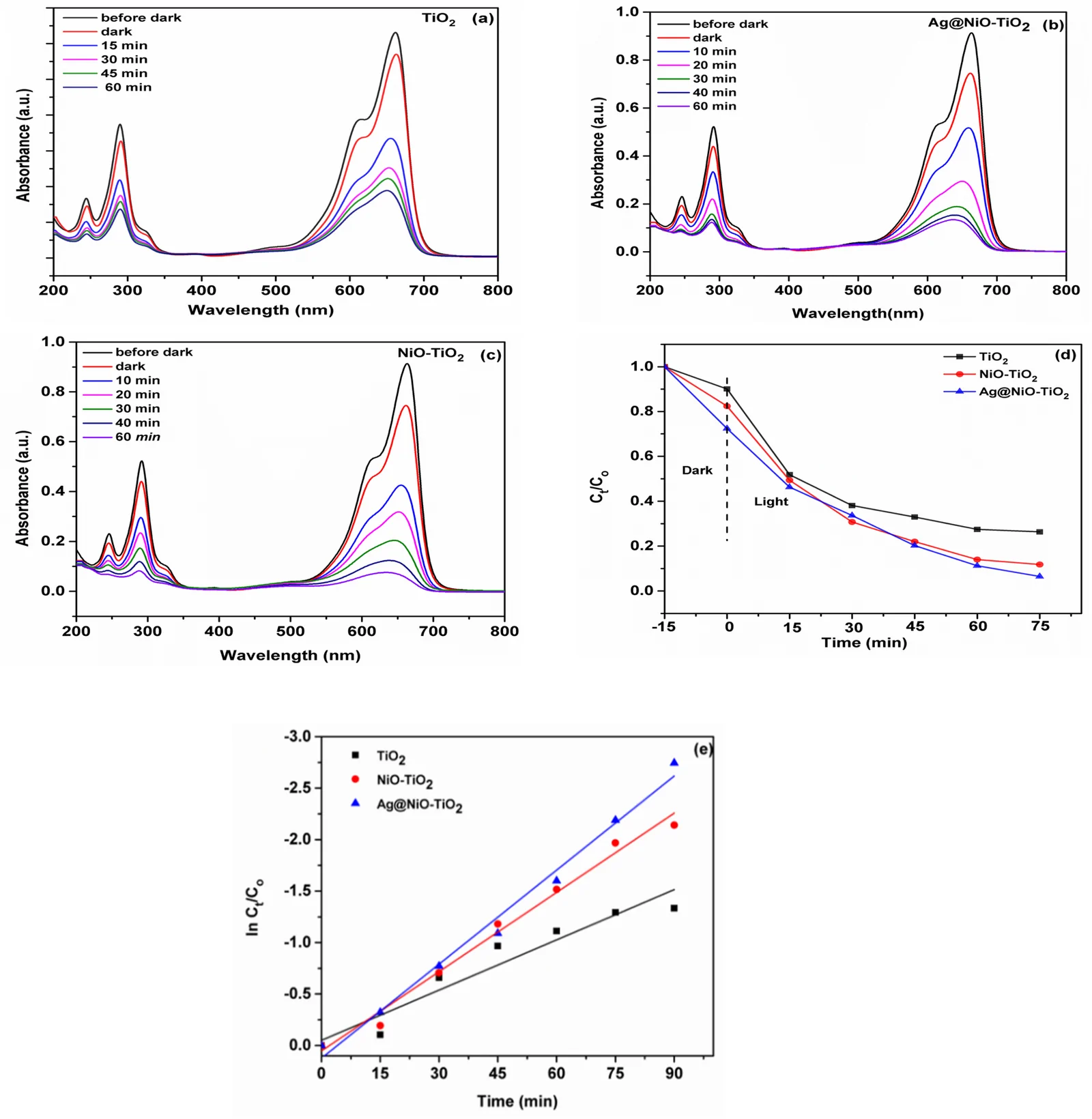
(a) UV-vis absorption spectra of an MB solution degradation with TiO_2_ nanoparticles. Photodegradation of MB solution by the (b) NiO–TiO_2_ and (c) Ag@NiO–TiO_2_ nanocomposites in 60 min under UV-visible light irradiation. (d) Plot of *C*_*t*_/*C*_0_ as a function of the irradiation time for the comparison of TiO_2_, NiO–TiO_2_ and the Ag@NiO–TiO_2_ nanocomposite. (e) Pseudo-first-order kinetics for the degradation of TiO_2_, NiO–TiO_2_ and the Ag@NiO–TiO_2_ nanocomposite (20 mg L^−1^).

Fig. 5d shows *C*_*t*_/*C*_0_ plotted *vs.* time, where *C*_*t*_ is the concentration following irradiation time *t* and *C*_0_ is the initial MB concentration following dark adsorption equilibrium. Higher degradation efficiency is indicated by a lower *C*_*t*_/*C*_0_. The Ag@NiO–TiO_2_ sample has the best MB removal performance since it exhibits the lowest final *C*_*t*_/*C*_0_. The kinetic curve of ln(*C*_*t*_/*C*_0_) with irradiation time is displayed in Fig. 5e. The almost linear relationship implies pseudo-first-order kinetics for MB degradation. Ag@NiO–TiO_2_ has the greatest reaction rate constant due to its steeper slope.^49^

#### 3.2.2. CV dye photodegradation

The photodegradation studies were carried out utilizing UV-visible light irradiation with photocatalysts, namely, TiO_2_, NiO–TiO_2_, and Ag@NiO–TiO_2_. To establish an adsorption–desorption equilibrium, the CV dye wastewater and a specific photocatalyst were stirred in the dark for 15 minutes before being exposed to UV-visible light. The initial dye concentration used in the experiments was 50 mg L^−1^, and the catalyst dose was 20 mg L^−1^ at the original pH 6.5 of dye solution. Fig. 6a–c display the results for the time-dependent dye degradation.

**Fig. 6:**
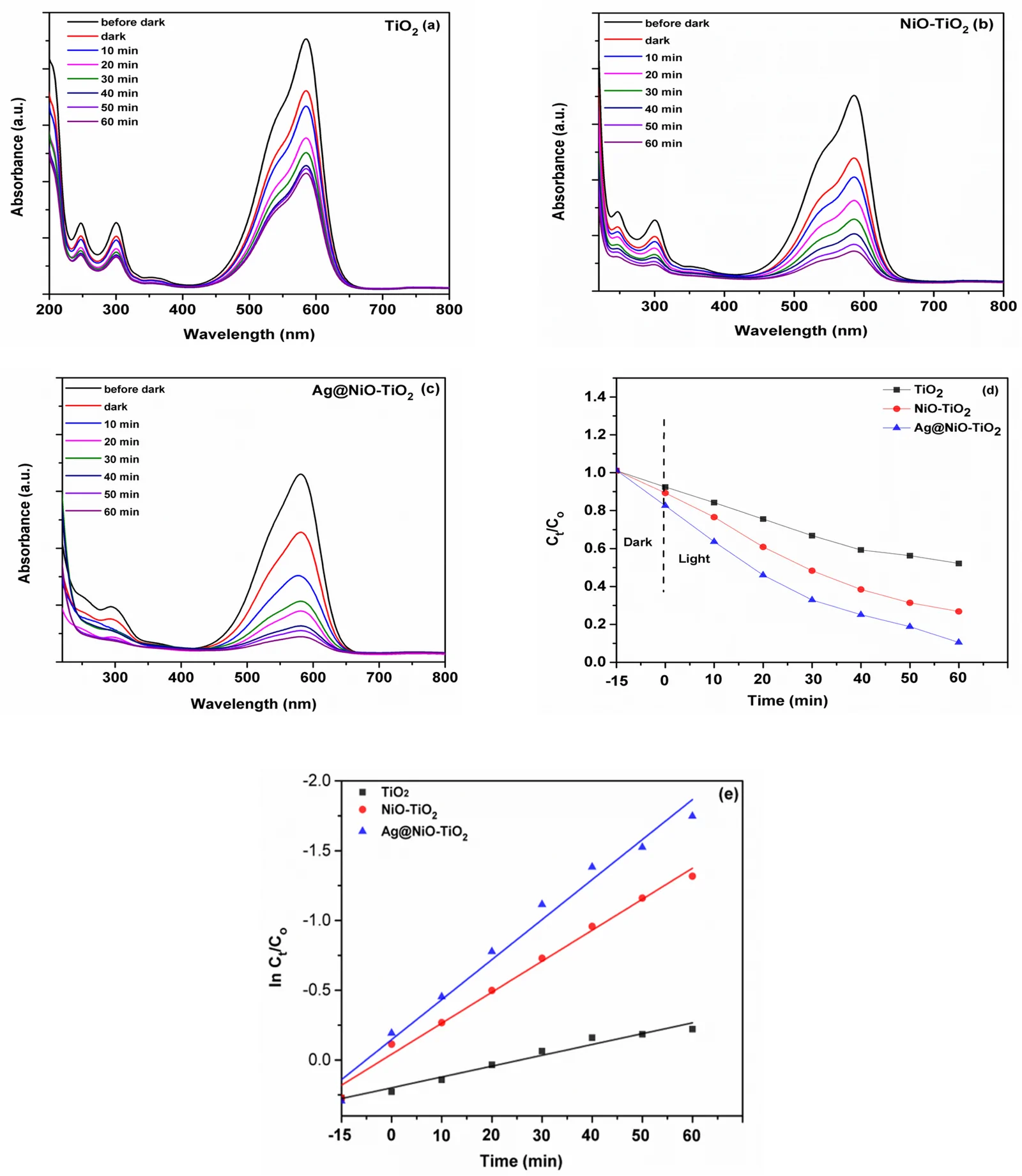
(a) UV-vis absorption spectra of a CV dye solution degradation with TiO_2_ nanoparticles under UV-visible light irradiation. Photodegradation of a CV dye solution by the (b) NiO–TiO_2_ and (c) Ag@NiO–TiO_2_ nanocomposites in 60 min under UV-visible light irradiation. (d) Plots of *C*_*t*_/*C*_0_ as a function of the irradiation time for the comparison of TiO_2_, NiO–TiO_2_ and the Ag@NiO–TiO_2_ nanocomposite. (e) Pseudo-first-order kinetics for the degradation of TiO_2_, NiO–TiO_2_ and the Ag@NiO–TiO_2_ nanocomposite (20 mg L^−1^).

During the entire span of 60-minute testing period, the blank photodegradation run with any catalyst showed little photodegradation performance. Dye degradation, however, varied significantly over time when all photocatalysts were present. The outcomes of the dark condition displayed the improved adsorption capacity of the Ag@NiO–TiO_2_ photocatalyst, which is essential for the efficient dye degradation under UV-visible light irradiation. The order of several photocatalysts photocatalytic activity was Ag@NiO–TiO_2_ > NiO–TiO_2_ > TiO_2_. After a 60-minute treatment period, the dye removal efficiencies for TiO_2_, NiO–TiO_2_, and Ag@NiO–TiO_2_ photocatalysts were calculated to be 48%, 62%, and 90%, respectively. The synergistic effect of TiO_2_ photocatalytic, NiO and Ag on dye adsorption capacity worked well and increased the photodegradation efficiency of the Ag@NiO–TiO_2_ composite. Fig. 6d displays *C*_*t*_/*C*_0_ plotted *vs.* time, where *C*_*t*_ is the concentration following irradiation time *t* and *C*_0_ is the initial CV dye concentration following dark adsorption equilibrium. Higher degradation efficiency is indicated by a lower *C*_*t*_/*C*_0_ value. The Ag@NiO–TiO_2_ sample has the best CV dye removal performance, as it shows the lowest final *C*_*t*_/*C*_0_ value.

The Langmuir–Hinshelwood model, which is generally used for the photocatalytic degradation of organic compounds, is utilized to evaluate the kinetics of dye degradation. The dependency of the photocatalytic reaction rate on the concentration of organic pollutants at low substance concentrations is represented by the pseudo-first-order equation (Fig. 6e).^18^ The known pseudo-first-order kinetics is given by eqn (2) as follows:

2 −ln(*C*_*t*_/*C*_0_) = *kt*.

The rate constant *k* for TiO_2_, NiO–TiO_2_, and Ag@NiO–TiO_2_ nanoparticles is 0.01114, 0.014123 and 0.03141 min^−1^ based on the plot of ln(*C*_*t*_/*C*_0_) *vs. t*. As shown in Fig. 6e, the photocatalytic degradation rate of CV with dark, catalyst and photolysis had a good linear correlation (*R*^2^) with time of 0.97032, 0.98983, and 0.97791, respectively. One feasible explanation for the significant improvement in the photodegradation efficiency of the Ag@NiO–TiO_2_ catalyst was that NiO and Ag improve the photogenerated electrons and holes and prevent the recombination process. Additionally, by effectively separating electrons and holes at the AgNiO–TiO_2_ interfaces, these materials prevent interfacial charge transfer processes.

#### 3.2.3. Ciprofloxacin antibiotic photodegradation

Fig. 7(a–c) display a comparative plot of relative concentration *vs.* irradiation duration after the UV-vis monitoring of ciprofloxacin (CIP) degradation using three photocatalysts: TiO_2_, NiO–TiO_2_, and Ag@NiO–TiO_2_. The strong UV absorption of ciprofloxacin is mostly observed around 275–278 nm, with additional bands and shoulders frequently observed at 316 and 331 nm. Since these bands originate from the fluoroquinolone chromophore, it is a routine practice to monitor CIP degradation by observing how they diminish under irradiation. The CIP concentration was measured by UV-vis spectroscopy at around 277 nm in several CIP photocatalysis investigations.^50^

**Fig. 7:**
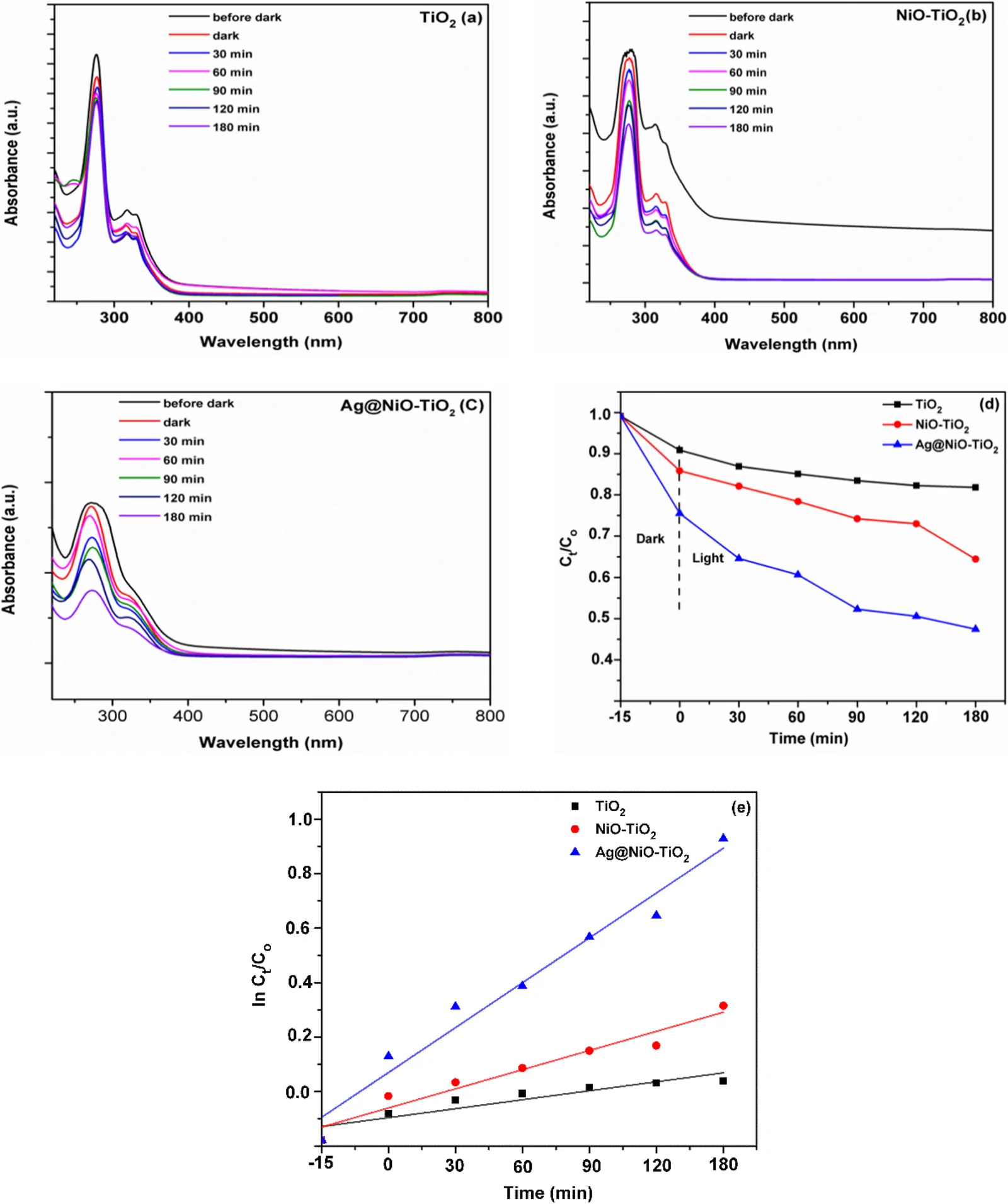
(a) UV-vis absorption spectra of ciprofloxacin solution degradation with TiO_2_ nanoparticles. Photodegradation of ciprofloxacin solution by the (b) NiO–TiO_2_ and (c) Ag@NiO–TiO_2_ nanocomposites in 60 min under UV-visible light irradiation. (d) Plots of *C*_*t*_/*C*_0_ as a function of the irradiation time for the comparison of TiO_2_, NiO–TiO_2_ and the Ag@NiO–TiO_2_ nanocomposite. (e) Pseudo first-order kinetics for the degradation of TiO_2_, NiO–TiO_2_ and the Ag@NiO–TiO_2_ nanocomposite (20 mg L^−1^).

TiO_2_, NiO–TiO_2_, and Ag@NiO–TiO_2_ absorbance spectra showed how the concentration of ciprofloxacin (CIP) varies during the photocatalytic degradation process. The dark spectrum is recorded after the catalyst and CIP solution are kept in the dark, frequently to assess the degree of adsorption on the catalyst surface, whereas the before dark spectrum represents the original CIP solution prior to adsorption equilibrium being established. The spectra obtained between 30 and 180 min represent varying light irradiation times. A drop in CIP concentration is indicated by a decrease in the distinctive CIP absorption peak, which is observed between 275 and 300 nm. Consequently, more effective photocatalytic degradation is suggested by a quicker and larger drop in this peak.

The major ciprofloxacin absorption peak in TiO_2_ (a) only marginally declines from the dark stage to 180 minutes of light irradiation in Fig. 7a, suggesting that bare TiO_2_ exhibits poor photocatalytic effectiveness under the studied environment. Despite TiO_2_ stability and widespread usage as a photocatalyst, its activity is limited by the fast recombination of photogenerated electron–hole pairs and its comparatively large band gap, which is typically between 3.0 and 3.2 eV. These findings weaken the photocatalytic efficiency and visible light usage.^51^ TiO_2_ can therefore produce reactive species, but before many of the photogenerated electrons and holes can react with oxygen, water, or hydroxide ions to efficiently breakdown ciprofloxacin, they recombine.

The ciprofloxacin absorption peak in Fig. 7b for NiO–TiO_2_ is clearly reduced compared to bare TiO_2_, suggesting that the inclusion of NiO enhances the photocatalytic degradation ability. The development of a NiO/TiO_2_ heterojunction, in which NiO functions as a p-type semiconductor and TiO_2_ typically operates as an n-type semiconductor, is most likely the cause of this increase. Combining these two materials can create a p–n junction, which encourages the separation of photogenerated electrons and holes and decreases their recombination. More electrons and holes can take part in surface reactions and produce reactive oxygen species such as hydroxyl radicals (˙OH), superoxide radicals (O_2_˙^−^), and photogenerated holes (h^+^) to improve charge separation. The piperazine ring, quinolone core, carboxyl group, and other active areas of ciprofloxacin molecules possibly will then be attacked by these reactive species.^50^

Similarly, ciprofloxacin absorbance peak in the presence of catalyst Ag@NiO–TiO_2_ in Fig. 7(c) lowers the greatest during 180 minutes of light irradiation, suggesting that Ag doping offers the best CIP degradation of the three catalysts. Ag plays many roles in the TiO_2_-based photocatalytic system, which accounts for the increased activity. By taking in photogenerated electrons, Ag can function as an electron trap, improving charge separation and lowering electron–hole recombination. Ag nanoparticles can also improve visible light absorption through surface plasmon resonance, which makes it possible for the catalyst to use light more efficiently. By helping as a bridge between NiO and TiO_2_, Ag may also enhance interfacial charge transfer by allowing charges to flow throughout the composite structure. Consequently, additional reactive oxygen species are created, and contribute to the breakdown of CIP, including hydroxyl radicals (˙OH) and superoxide radicals (O_2_˙^−^).^51^ This behavior is in line with the findings that show how doping, metal decorating, and heterojunction building improve light absorption, charge separation, and reactive species formation to increase ciprofloxacin photocatalytic degradation.

By plotting the relative ciprofloxacin concentration, *C*_*t*_/*C*_0_, *versus* irradiation time, where *C*_0_ is the starting CIP concentration and *C*_*t*_ is the CIP concentration at a certain time, as shown in Fig. 7d, the degradation performance of the three catalysts can be quantitatively evaluated. However, the general pattern is evident because higher CIP removal is indicated by a lower *C*_*t*_/*C*_0_ number. After 180 minutes, the TiO_2_ curve showed the lowest degradation efficiency, declining slowly, whereas the NiO–TiO_2_ curve shows a more pronounced decline. On the other hand, Ag@NiO–TiO_2_, exhibits the greatest degradation efficiency after 180 minutes. Therefore, Ag@NiO–TiO_2_ > NiO–TiO_2_ > TiO_2_ is the order of photocatalytic activity, which is consistent with the UV-vis spectra, where Ag@NiO–TiO_2_ shows the largest decrease in CIP absorbance peak. Fig. 7e illustrates the plot of ln(*C*_0_/*C*_*t*_) against irradiation time and the photocatalytic degradation kinetics of ciprofloxacin utilizing TiO_2_, NiO–TiO_2_, and Ag@NiO–TiO_2_. Greater ciprofloxacin degradation is indicated by a larger ln(*C*_0_/*C*_*t*_) value, where *C*_0_ is the starting ciprofloxacin concentration and *C*_*t*_ is the concentration at time *t*. The decline appears to follow a pseudo-first-order kinetic model, based on the roughly linear rise over time. TiO_2_ has the lowest ln(*C*_0_/*C*_*t*_) value and the smallest slope among the three catalysts, suggesting the slowest rate of degradation because of reduced photocatalytic activity and restricted charge separation. The greater slope of NiO–TiO_2_ compared to TiO_2_ confirms that NiO increases the rate of degradation by creating a heterojunction that facilitates electron–hole separation. After 180 minutes, Ag@NiO–TiO_2_ shows the steepest slope and the highest ln(*C*_0_/*C*_*t*_) value, indicating the quickest and most effective ciprofloxacin degradation. Ag decoration, which may boost visible light absorption, trap photogenerated electrons, decrease recombination, and encourage the creation of reactive oxygen species, is primarily responsible for this enhancement.

### 3.3. Antibacterial and antifungal activities

The antibacterial properties of TiO_2_, NiO–TiO_2_, and Ag@NiO–TiO_2_ nanoparticles against *Enterobacter aerogenes*, *Salmonella typhimurium*, and *Bordetella bronchiseptica* were evaluated *in vitro*. The antifungal activity was tested using *Aspergillus fumigatus*, *Fusarium solani* and *A. niger*. Table 1 shows the *in vitro* antibacterial findings. With a few exceptions, Ag@NiO–TiO_2_ displayed antibacterial effectiveness against the chosen microorganisms, but TiO_2_ was least effective against any type of bacteria or fungi. NiO–TiO_2_ and Ag@NiO–TiO_2_ test compounds have demonstrated a greater activity against *Enterobacter aerogenes*, *Salmonella typhimurium*, and *Bordetella bronchiseptica* than those of the conventional medication Cefixime. These positive findings make compounds NiO–TiO_2_ and Ag@NiO–TiO_2_ effective against *Enterobacter aerogenes*, *Salmonella typhimurium*, and *Bordetella bronchiseptica*, and more research is required. The previously observed antibacterial activity pattern of metal oxide nanoparticles was supported by the increased antifungal activity of Ag@NiO–TiO_2_ compared to TiO_2_ and its derivatives.^52^ Comparable antibacterial activity was demonstrated by the test compounds and structurally similar TiO_2_-based compounds that have been previously described.

**Table 1:** Results of the zone of inhibition of bacteria by TiO_2_, NiO–TiO_2_, Ag@NiO–TiO_2_ and cefixime

S. no.	Bacterial species	Zone of inhibition (mm)			
		TiO_2_	NiO–TiO_2_	Ag@NiO–TiO_2_	Cefixime
1	*Enterobacter aerogenes*	10 ± 1.89	15 ± 1.23	18 ± 1.81	25 ± 1.11
2	*Salmonella typhimurium*	13 ± 1.62	15.5 ± 1.71	19 ± 1.27	24 ± 1.77
3	*Bordetella bronchiseptica*	12 ± 1.51	14 ± 1.14	15 ± 1.82	24.5 ± 1.71

It is noted that Ag@NiO–TiO_2_ NPs effectively inhibited the growth of all three G-ve bacteria at relatively low doses, as indicated by their low MIC values of 6.25 µg mL^−1^. Therefore, Ag@NiO–TiO_2_ NPs showed strong bactericidal action against these bacteria with MBC values of 6.25–12.5 µg mL^−1^. The capacity of Ag@NiO–TiO_2_ nanoparticles to show bactericidal action against all three G-ve pathogens was proven by an MBC/MIC ratio of 1–2 (Table 2). Ag@NiO–TiO_2_ NPs are very efficient in inhibiting and killing *E. aerogenes* at relatively low doses, as indicated by their lower MIC value of 6.25 µg mL^−1^ and lower MBC value of 6.25 µg mL^−1^. The bactericidal activity of Ag@NiO–TiO_2_ NPs against *E. aerogenes* was validated by an MBC/MIC ratio of 1.

**Table 2:** MIC and MBC of TiO_2_, NiO–TiO_2_ and Ag@NiO–TiO_2_ against various tested bacterial strains

S. no.	Compound	*E. aerogenes*		*S. typhimurium*		*B. bronchiseptica*	
		MIC (µg mL^−1^)	MBC (µg mL^−1^)	MIC (µg mL^−1^)	MBC (µg mL^−1^)	MIC (µg mL^−1^)	MBC (µg mL^−1^)
1	TiO_2_	50	100	50	100	25	100
2	NiO–TiO_2_	25	50	12.5	25	12.5	50
3	Ag@NiO–TiO_2_	6.25	6.25	6.25	12.5	6.25	12.5

The antifungal activities of TiO_2_, NiO–TiO_2_, and Ag@NiO–TiO_2_ were tested against three strains (*Aspergillus fumigatus*, *Fusarium solani*, and *A. niger*) using the standard protocol reported in the literature.^31^ The results are shown in Table 3. In general, Ag@NiO–TiO_2_ showed greater antifungal activity than the corresponding TiO_2_ and NiO–TiO_2_ against all three strains. The structural variety of cells in both types of microorganisms is responsible for the notable differences in activity patterns between antibacterial and antifungal agents. Here, the Ag@NiO–TiO_2_ nanocomposite showed the highest activity against the three types of fungus. The supplemental data in Table 3 contain the antifungal data values together with the SD (standard deviation). Table 4 summarizes the available literature studies on the comparison of the performance of the Ag@NiO–TiO_2_ composite with that of the available TiO_2_-based photocatalytic systems. TiO_2_-based semiconductor composites outperformed other materials in terms of photocatalytic activity. The Ag@NiO–TiO_2_ NPs need to be evaluated as the photocatalyst and antimicrobial agent by further studies.

**Table 3:** Results of the zone of inhibition of fungi by TiO_2_, NiO–TiO_2_, Ag@NiO–TiO_2_ and terbinafine

S. no.	Fungal species	Zone of inhibition (mm)			
		TiO_2_	NiO–TiO_2_	Ag@NiO–TiO_2_	Terbinafine
1	*Aspergillus fumigatus*	7.5 ± 0.707	13.5 ± 1.41	19 ± 1.141	22 ± 1.11
2	*Fusarium solani*	12.5 ± 1.12	14 ± 0.707	20 ± 1.12	22 ± 1.55
3	*A. niger*	10 ± 1.21	11 ± 1.41	17.5 ± 1.11	24 ± 1.71

**Table 4:** Comparison with previously described photocatalytic/antimicrobial TiO_2_-based heterojunctions

TiO_2_-based heterojunction	Target pollutant	Photocatalytic activity	Light/time	Anti-microbial activity	Main distinction relative to Ag@NiO–TiO_2_	Ref.
NiO/Ag/TiO_2_ ternary heterojunction	MB	93%	UV; 90 min	Not evaluated	Only focused on MB photodegradation rather than the combined MB–CV–CIP–antimicrobial scope	53
ZnO/TiO_2_ direct Z-scheme	MB	88%	UV; 90 min	Significant inhibition against *S. aureus*, *K. pneumoniae* and *P. aeruginosa* at 150 µg mL^−1^	Multifunctional dye degradation/antibacterial system, but only 88.9% MB and no CIP/CV assessment	54
Fe_3_O_4_/rGO/TiO_2_	MB	≈100%	4 min	4.92-Log reduction of *E. coli* in 60 min	Fast MB degradation and strong bacterial inactivation	55
TiO_2_/GO heterojunction	CV	96%	Visible light, 90 min; 10 ppm CV, pH 8, 0.02 g catalyst	Not reported	Strong CV benchmark but lacks antibiotic degradation and antibacterial multifunctionality	56
	MB	≈100% at optimum pH	150 min; halogen illumination, pH ≈ 11.8	MIC 0.195 µg mL^−1^ against *S. aureus*; after *γ* irradiation ZOI up to 44 mm and MIC 0.024 µg mL^−1^	Very strong antimicrobial response but substantially more complex composition/post-treatment and strongly alkaline optimum for MB degradation	57
TiO_2_/CuO p–n heterojunction		Primarily antimicrobial study	Visible light	*E. coli*: 96.2% inactivation in 10 min; 100% in 20 min; 64.7% in dark at 10 min	Excellent antibacterial benchmark and another p–n TiO_2_ heterojunction, but lacks demonstrated simultaneous organic-pollutant degradation breadth	32
CeO_2_–TiO_2_ heterojunction	MB	96%	Visible light	Not reported	High MB efficiency and green synthesis; no antibacterial evaluation	58
CeO_2_–TiO_2_ heterojunction	CIP	93%	Visible light	Not reported	Particularly useful recent CIP benchmark; demonstrates MB + CIP but not CV or antimicrobial activity	58
5% Cu_2_O–TiO_2_ p-n heterojunction	CIP	96.6%; *k* = 0.03005 min^−1^	Visible light, 110 min; 10 ppm CIP	Not reported	Strong CIP benchmark; no antimicrobial/dye-spectrum validation	59
Zn-doped TiO_2_	MB and CV	98%	Natural sunlight	Not central to study	Useful multi-dye benchmark; doped binary rather than plasmonic ternary; no CIP	60
TiO_2_/Sn_2_S_3_	CV	75.93%	60 min	Not reported	Lower CV benchmark and single-pollutant demonstration	61
TiO_2_–MgO/kaolinite	CV	99.8% removal	UV irradiation	Not reported	Very high CV removal; adsorption by clay support may contribute; no CIP/antimicrobial function	62
Ag@NiO–TiO_2_	MB	88%	UV-visible; 60 min	*Enterobacter aerogenes*; ZOI up to 18 mm/MIC 6.25 µg mL^−1^/75% inhibition	Plasmonic Ag + p-type NiO + n-type TiO_2_; same material evaluated for dyes, antibiotic and antimicrobial activity	This work
Ag@NiO–TiO_2_	Crystal violet (CV)	90%	UV-visible; 60 min	*Salmonella typhimurium*; ZOI up to 19 mm/MIC 6.25 µg mL^−1^ and 80% inhibition	Extends activity to a triphenyl-methane dye in addition to MB	This work
Ag@NiO–TiO_2_	Ciprofloxacin (CIP)	57%	UV-visible; 180 min	*Bordetella bronchiseptica*; ZOI up to 15 mm/MIC 6.25 µg mL^−1^ and 60% inhibition	Demonstrates pharmaceutical pollutant degradation in addition to dye removal	This work

The above literature review showed that TiO_2_-based nanocomposites are effective photocatalysts and antimicrobial agents though not unique in novelty, but the Ag/NiO/TiO_2_ ternary composition is unique in and of itself. In 2023, a ternary NiO/Ag/TiO_2_ heterojunction was reported to degrade methylene blue and medicinal chemicals under visible light, achieving 93.15% MB degradation in 60 minutes. Therefore, the stronger novelty position is that the current Ag@NiO–TiO_2_ system is assessed as a single multifunctional platform across structurally distinct dyes, an antibiotic pollutant and microorganisms, especially for crystal-violet, ciprofloxacin, and antimicrobial experiments that have not been previously reported for this particular Ag@NiO–TiO_2_ architecture.

### 3.4. MB, CV and CIP mineralization

The total organic carbon (TOC) indicates the degree of MB, CV and CIP mineralization. To determine the mineralization of the MB dye, CV dye and CIP antibiotic using the Ag@NiO–TiO_2_ NPs, TOC tests were carried out. A significant decrease in TOC was found, indicating effective MB, CV and CIP mineralization. The mineralization process of MB, CV and CIP under ideal photocatalytic conditions was examined using TOC analysis. After 1 h of the reaction, the findings displayed TOC removal efficiencies for MB, CV and CIP to be 82%, 65% and 47%, respectively. The formation of persistent intermediates during the degradation processes, each of which follows a different degradation pathway, may be the cause of the difference in mineralization and degradation rates.

Since only the carbon part of each molecule contributes to the TOC measurement, even if the initial concentration of each chemical was 50 ppm, this number does not precisely correlate to 50 ppm TOC. The theoretical initial TOC concentrations for MB, CV and CIP were around 60.1, 73.6 and 61.6 ppm, respectively, based on their molecular compositions. The residual TOC values after treatment were 11.10 ppm for MB, 26.24 ppm for CV, and 31.81 ppm for CIP. These numbers translate into projected mineralization efficiencies around 82.0%, 64.3% and 47.1%, respectively. As a result, MB showed the highest level of mineralization, followed by CV and CIP.

### 3.5. Reusability and stability of photocatalysts

The ideal parameters for the photocatalyst Ag@NiO–TiO_2_ with a catalyst dose of 0.03 g, a pH of 9, and a concentration of 20 ppm were tested for the reusability and stability. After the tests, the sample was collected and dried in an oven at 70 °C. Even after four cycles, there were no substantial variations in the photocatalyst performance, as shown in Fig. 8a. This experiment showed that the Ag@NiO–TiO_2_ photocatalyst has excellent recyclability and stability, and may be effectively employed in the degradation of organic waste. From the first to the fourth cycle, there is only a little drop in degradation efficiency. CIP degradation decreases from around 64% to 52%, CV degradation from roughly 97% to 88–89%, and MB degradation from 95% to about 82%, whereas the reduction in degradation may result from the catalyst loss during recovery, partial blocking of active sites by adsorbed intermediates, or photocatalyst deactivation following repeated uses. This retention of activity indicates a high photocatalytic stability and recyclability. Reusability is a crucial factor for effective photocatalytic wastewater treatment because catalyst recovery and activity retention have a direct impact on process practicality.^63^

**Fig. 8:**
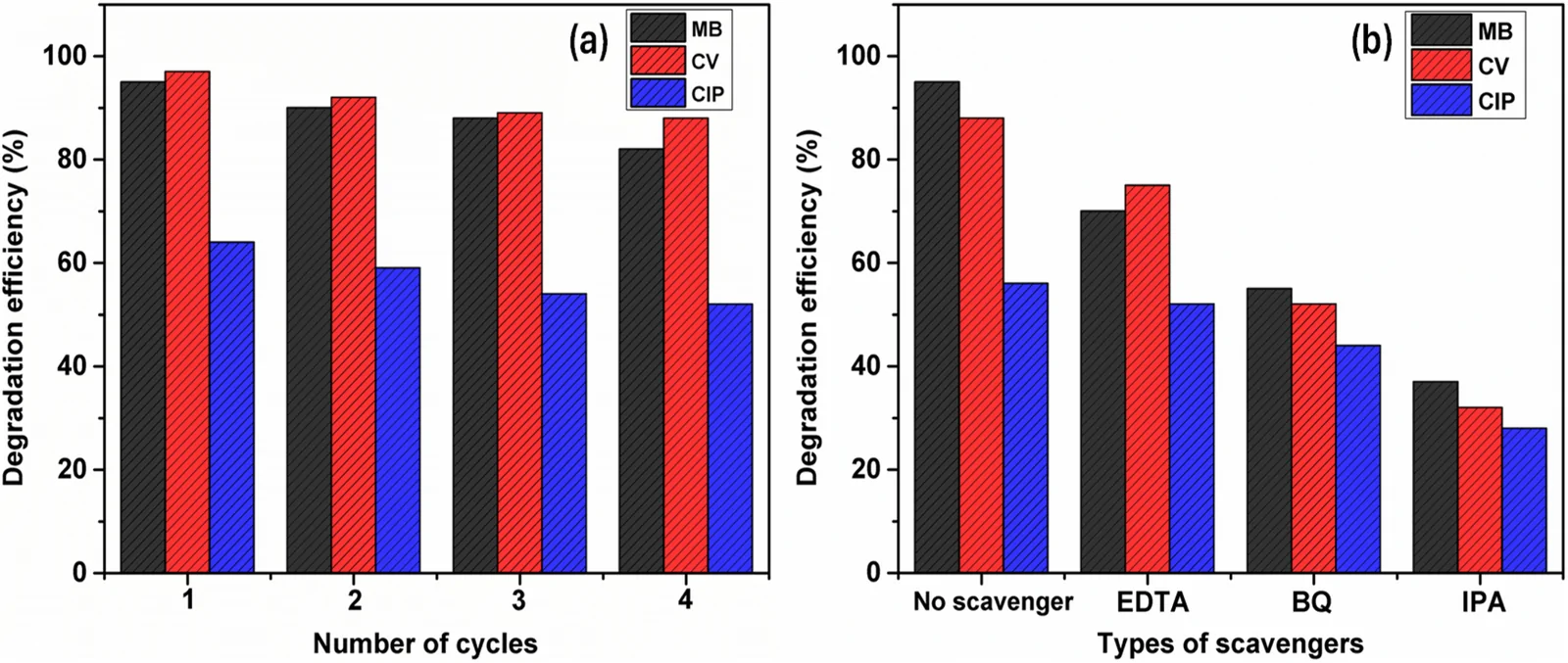
(a) Reusability and photocatalyst stability of Ag@NiO–TiO_2_ on MB, CV and CIP degradation. (b) Trapping tests showing the influence of scavengers on MB, CV and CIP dye degradation.

### 3.6. Radical trapping experiment

The impact of several radical scavengers on photocatalytic degradation is displayed in Fig. 8b. The highest degradation efficiency is achieved when scavengers are not present. Prior to experiments, 1 mM scavenger was added to MB, CV and CIP solutions. To achieve the adsorption–desorption equilibrium between the photocatalyst and the dye, the stirring is conducted in the dark for 15 min. The concentration of MB, CV and CIP was measured after the solution was collected at 15 min intervals following irradiation by simulated solar light. The addition of EDTA resulted in a minor reduction, while BQ showed a higher inhibition. When IPA is present, the decrease in degradation is most noticeable. BQ captures superoxide radicals (O_2_˙^−^), IPA scavenges hydroxyl radicals (OH˙), and EDTA is frequently utilized as a scavenger for photogenerated holes (h^+^). As a result, the significant suppression caused by IPA indicates that OH˙ radicals are crucial to the photocatalytic degradation process, while O_2_˙^−^ and h^+^ also contribute to the degradation of pollutants. These ROS species can promote the breakdown of pollutants. They can also aid in the inactivation of bacteria by causing damage to intracellular components, proteins, and cell walls/membranes.

In addition, Fig. 9 shows the FTIR spectrum, XRD pattern, SEM images, and SEM-EDS spectrum of the recycled photocatalyst after four cycles. All the sample functional groups were visible during photocatalyst regeneration, and its molecular structure was maintained, according to the FT-IR result shown in Fig. 9b. The XRD result in Fig. 9b indicates that the reused sample crystallinity is maintained. Neither a new peak nor an impurity was visible. The SEM results in Fig. 9c and d show that the photocatalyst morphology remains unchanged. Even after four cycles, the distribution in Fig. 9e shows that all the elements are evenly distributed and that no extra materials are observed.

**Fig. 9:**
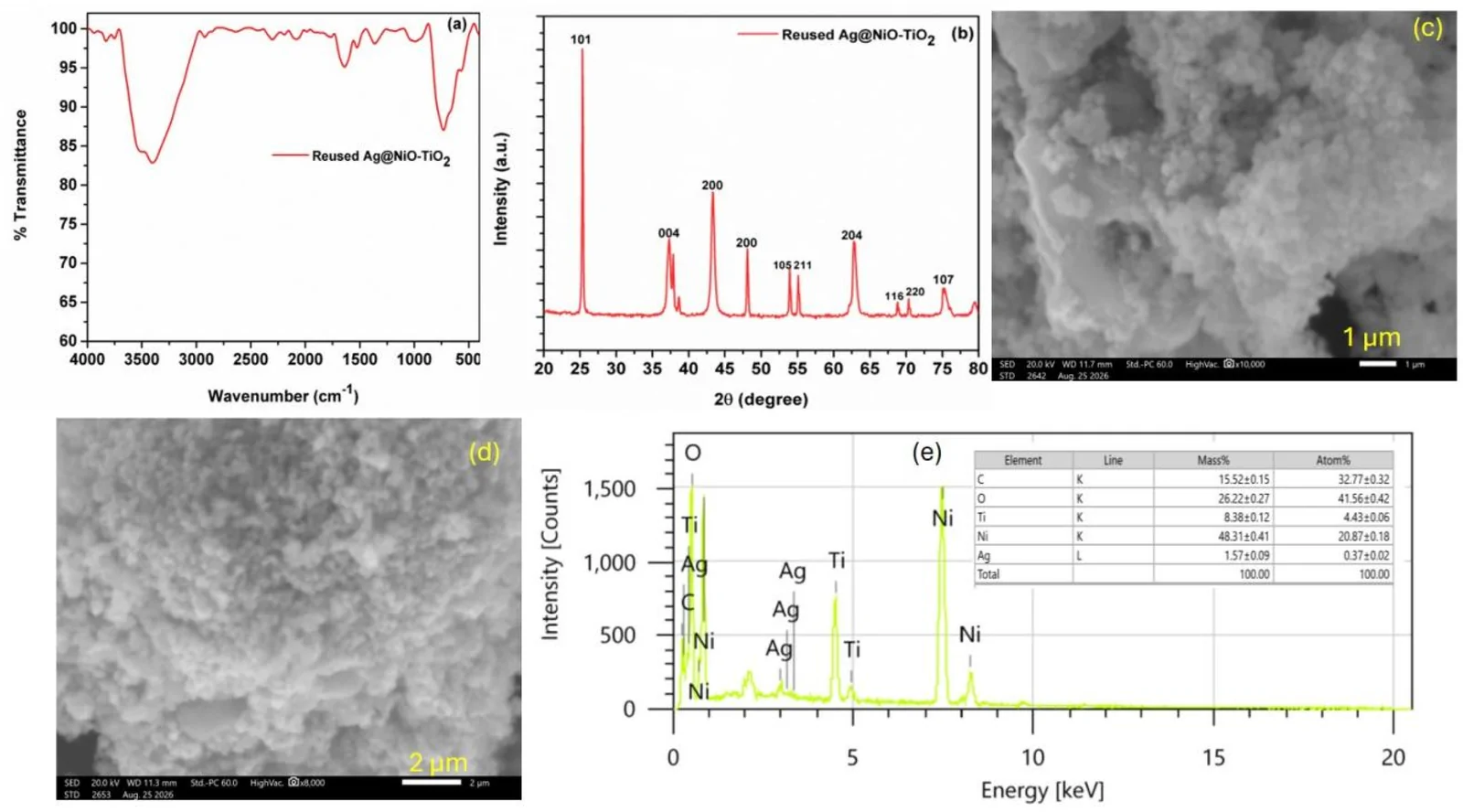
(a) XRD pattern, (b) FTIR spectrum, (c and d) SEM images and (e) EDS spectrum (Ti, O, Ni, and Ag elements) of the reused composite after four cycles.

The current photocatalytic degradation studies were carried out in a controlled environment with synthetic single-pollutant solutions. Real industrial wastewater contains a variety of inorganic ions and dissolved organic matter that may affect the photocatalytic efficiency, even though our studies shed light on the intrinsic photocatalytic performance of the given materials. The degradation rate may be altered by species like Cl^−^, HCO_3_^−^, SO_4_^2−^, NO_3_^−^, and natural organic matter that compete with target pollutants for adsorption sites or interact with photogenerated reactive species. Specifically, extremely reactive hydroxyl radicals can be scavenged by bicarbonate and natural organic materials, which could decrease the photocatalytic efficacy.^64^ Therefore, to assess the prepared photocatalyst practical use under complex environmental conditions, future research studies would be carried out with real or suggested industrial wastewater matrices.

### 3.7. Effect of pH, initial concentration of MB, CV and CIP solutions and catalyst dose on degradation

Using 10 mg per L MB dye, CV dye and CIP antibiotic as the starting concentration and 0.02 g of the Ag@NiO–TiO_2_ NPs as the photocatalyst quantity, the impact of pH on the photocatalytic activity of Ag@NiO–TiO_2_ NPs was examined between pH 3.0 and 11.0. The pH of 10 mg per L MB dye, CV dye and CIP antibiotic was determined to be approximately 6.5, and the pH of this solution was adjusted by using 1 mol per L NaOH or 1 mol L solution. Under UV-vis illumination, the percentage degradation *versus* time for different pH levels of 10 mg per L MB and CV solutions was measured. At pH 9, the greatest percentage of degradation was achieved in 60 minutes. The Ag@NiO–TiO_2_ surface charge is known to be pH sensitive, with a negative surface charge at pH 9. Therefore, the Ag@NiO–TiO_2_ NP composite surface charge is negative. MB and CV are cationic dyes, so the dye and the nanoparticles have strong electrostatic contact. The photocatalytic effect of Ag@NiO–TiO_2_ NPs was further initiated by this interaction. Similarly, the highest performance was observed for CIP degradation with Ag@NiO–TiO_2_ at a pH around 7.5, frequently near pH 7, rather than very alkaline pH like 9–11.

The effect of the initial dye concentration of 10, 30, 40 and 50 mg L^−1^ with the photocatalyst quantity at 0.02 g and the pH of the MB and CV solution at 9 was studied. It was experimentally observed that the percentage of dye removal decreases from 10 to 50 mg L^−1^. The parts of the Ag@NiO–TiO_2_ NP surface that are activated by light may be blocked by methylene blue and crystal violet dye molecules. As a result, it may be possible to stop the production of ˙OH radicals, which oxidize and destroy the color. Similar results for CIP degradation at pH 7.5 and the same conditions were observed.

Ag@NiO–TiO_2_ NPs with various concentrations in the range of 10–50 mg L^−1^ were used to degrade 10 ppm of MB and CV solution at a pH of 9.5 with a contact period of 60 min in order to investigate the impact of loading ability on the dye degradation efficiency. When the dose of Ag@NiO–TiO_2_ NPs was increased up to 30 mg L^−1^, the percentage of dye photodegradation also increased; after that, it became nearly constant. The overall number of active sites on the catalyst surface increases somewhat with the concentration of Ag@NiO–TiO_2_ NPs. Therefore, for 30 mg of catalyst, the maximum degradation for MB and CV is observed for 10 ppm solution of MB and CV. Ag@NiO–TiO_2_ NPs nanocomposites (0.02 g L^−1^) under constant conditions were used to examine the impact of initial ciprofloxacin (CIP) concentration (10 ppm, 20 ppm, 30 ppm, 40 ppm and 50 ppm) on the photocatalytic performance. The degradation efficiency varies greatly with the starting CIP concentration. After 180 minutes of irradiation, the greatest degradation of 64% was achieved at 10 ppm, compared to 57.2%, 48%, 42% and 36% for 20 ppm, 30 ppm, 40 ppm and 50 ppm solutions of the Ag@NiO–TiO_2_ composite, respectively, as the most efficient catalyst in all.

### 3.8. Mechanism of the photocatalytic degradation of the Ag@NiO–TiO_2_ nanocomposite

The photodegradation pathway depends on OH˙ or O_2_˙^−^ radicals breaking down the organic pollutant into hazardous chemicals such CO_2_, H_2_O, nitrate, ammonium, and sulfate ions, as shown in eqn (3)–(6) as follows:^65^

3 Ag@NiO–TiO_2_ + Visible light → e^−^ + h^+^,

4 e^−^ + O_2_ → O_2_˙^−^,

5 h^+^ + H_2_O/OH^−^ → OH˙,

6 O_2_˙^−^/OH˙ + organic pollutants → intermediates → CO_2_ + H_2_O + non-toxic products.

The photocatalytic process for ciprofloxacin degradation over the Ag@NiO–TiO_2_ nanocomposite is also depicted in the graphical abstract (Fig. 10). The Ag@NiO–TiO_2_ catalyst produces photogenerated electrons and holes in the semiconductor components when exposed to visible light. A p–n heterojunction is created when p-type NiO and n-type TiO_2_ are coupled, which effectively separates electrons and holes and decreases their recombination. By enhancing visible light absorption through the surface plasmon resonance effect and serving as electron traps that promote interfacial charge transfer between NiO and TiO_2_, Ag nanoparticles further improve the process. Superoxide radicals (O_2_˙^−^) are created when separated electrons combine with dissolved oxygen, whereas hydroxyl radicals (˙OH) are created when photogenerated holes react with water or hydroxide ions.^66^ Ciprofloxacin molecules are attacked by these reactive oxygen species and holes at active locations such as the piperazine ring, quinolone core, carboxyl group, fluorinated ring, and cyclopropyl group. This results in degradation intermediates and ultimately mineralization into CO_2_, H_2_O, and inorganic ions. Overall, the figure shows how charge separation, radical production, and photocatalytic degradation efficiency are improved by Ag decoration and NiO–TiO_2_ heterojunction formation.^67^

**Fig. 10:**
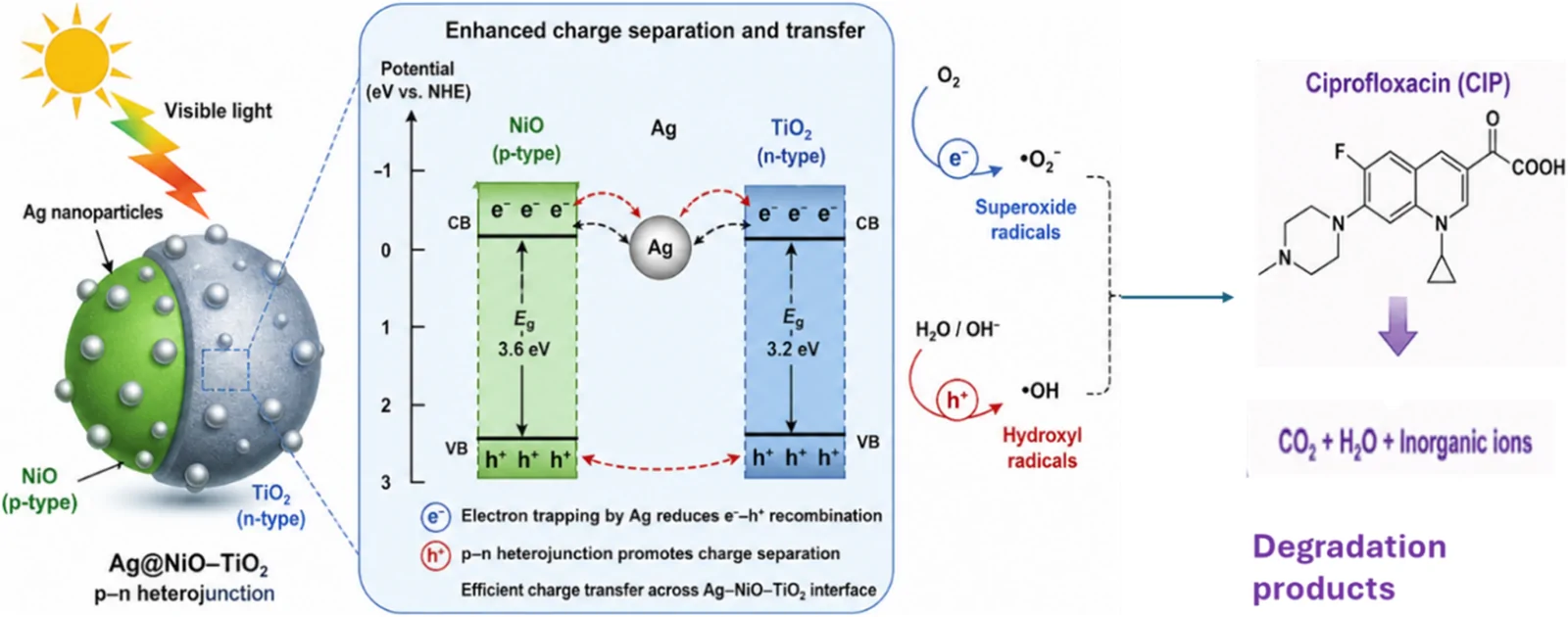
Photocatalytic mechanism of ciprofloxacin degradation over the Ag@NiO–TiO_2_ nanocomposite.

## 4. Conclusion

In conclusion, the Ag@NiO–TiO_2_ ternary heterojunction nanocomposite was effectively created by using a hydrothermal technique to evenly deposit and distribute NiO and Ag nanoparticles in sphere shape with diameters less than 10 nm on the surface of TiO_2_ with a size range of 100–200 nm. The material prepared was characterized using FT-IR spectroscopy, XRD, Raman spectroscopy, UV-visible spectroscopy, SEM-EDS mapping, HRTEM, TGA, CV and EIS. On the TiO_2_ surface, the Ag and NiO nanoparticles are evenly distributed with no other phase. In comparison to the other compounds (NiO–TiO_2_ and bare TiO_2_), the larger porosity and decreased band gap caused a higher wavelength shift in the UV-absorption spectra and a high generation of radicals, due to which Ag@NiO–TiO_2_ exhibited efficient photocatalytic activity. Ag@NiO–TiO_2_ showed outstanding organic compound photocatalytic degradation efficiency against MB, CV and CIP. Ag NPs considerably increase the catalytic activity and facilitate electron transfer by adding more electrons to the CB of TiO_2_, hence decreasing the electron–hole recombination. By lowering the energy band gap, moving the absorption to the visible light region, and improving the electron–hole pair separation efficacy, the close contact of TiO_2_, NiO, and Ag through the creation of Schottky junctions at the Ag–TiO_2_ and Ag–NiO interfaces and the formation of a p–n junction between TiO_2_ and NiO within the ternary nanocomposite structure all play critical roles in the photocatalytic activity. This study demonstrates how well the photocatalyst breaks down organic molecules. The antimicrobial activity showed that Ag@NiO–TiO_2_ is a good agent exhibiting high biocidal activity against all strains compared to TiO_2_ and NiO–TiO_2_ compounds. To assess the toxicity of the ternary Ag@NiO–TiO_2_ nanocomposite against microorganisms (such as bacteria and viruses), more research is needed.

## Conflicts of interest

There are no conflicts to declare.

## Data Availability

The data supporting the findings of this study are already provided in the manuscript. Additional data supporting the findings of this work are available from the corresponding author upon reasonable request.
